# Polymer-Directed Self-Assembly of BCNO Nanoparticles for Efficient Boron Utilization in Boron Neutron Capture Therapy

**DOI:** 10.1021/acsnanoscienceau.6c00088

**Published:** 2026-09-26

**Authors:** Wei-Hsin Chan, Lu-Ang Chen, Wei-Yuan Huang, Lin-Sheng Yu, Tzu-Chieh Sun, Fang-Hsin Chen, Chi-Shuo Chen, Tzu-Wei Wang, Pei-Yuin Keng

**Affiliations:** † Department of Materials Science and Engineering, 34881 National Tsing Hua University, Hsinchu City 300, Taiwan; ‡ Department of Biomedical Engineering and Environmental Sciences, National Tsing Hua University, Hsinchu City 300, Taiwan; § Boron Neutron Capture Therapy (BNCT) center, National Tsing Hua University, Hsinchu City 300, Taiwan; ∥ Institute of Nuclear Engineering and Science, National Tsing Hua University, Hsinchu City 300, Taiwan; ⊥ Institute of Oral Biology, National Yang Ming Chiao Tung University, Taipei 112304, Taiwan

**Keywords:** amphiphilic block copolymer, self-assembly, stimuli-responsive nanomaterial, boron neutron capture therapy (BNCT), boron nanoparticle, nanodrug, tumor spheroids

## Abstract

Boron carbon oxynitride (BCNO) nanoparticles are attractive boron-rich agents for boron neutron capture therapy (BNCT), yet their polymer-directed self-assembly, structural regulation, and delivery–function relationships remain insufficiently explored. Here, we report a stimuli-responsive polymer–BCNO nanoassembly, mPEG/PS-*b*-PEI@BCNO, that integrates macromolecular self-assembly with efficient boron utilization for BNCT against pancreatic cancer. The amphiphilic mPEG/PS-*b*-PEI copolymer was constructed by coupling methoxy poly­(ethylene glycol) (mPEG), polystyrene (PS), and poly­(ethylenimine) (PEI) through acid-labile Schiff-base linkages. Electrostatic complexation between negatively charged BCNO nanoparticles and cationic PEI, combined with solvent-driven amphiphilic assembly, generated spherical nanoassemblies with tunable morphology, size, and surface charge. Systematic variation of the mPEG/PS ratio revealed that the hydrophobic–hydrophilic balance of the copolymer governs BCNO packing and nanoassembly formation. The optimized mPEG/PS-*b*-PEI@BCNO nanoassemblies exhibited a uniform diameter of approximately 61 nm, positive surface charge, PEG-mediated colloidal stabilization, and acid-triggered disassembly into smaller BCNO-containing fragments under tumor-relevant acidic conditions. Despite a relatively low BCNO content of approximately 8 wt %, mPEG/PS-*b*-PEI@BCNO markedly improved boron delivery in UN-KC-6141 pancreatic cancer cells; the nanoassemblies achieved higher boron uptake efficiency than bare BCNO and boronophenylalanine (BPA). In 3D pancreatic tumor spheroids, mPEG/PS-*b*-PEI@BCNO delivered boron concentrations above the therapeutic threshold for BNCT and exhibited boron utilization efficiencies of 16.64% at pH 7.4 and 10.89% at pH 6.5. Under equivalent ^10^B dosing, the nanoassemblies produced the strongest BNCT response in 3D spheroids, inducing approximately 52% cell death and significantly outperforming bare BCNO and BPA. These findings demonstrate that polymer-controlled nanoassembly architecture enhances boron utilization without relying solely on high intrinsic boron loading, establishing a design framework for the next-generation BNCT nanodrugs.

## Graphical abstract

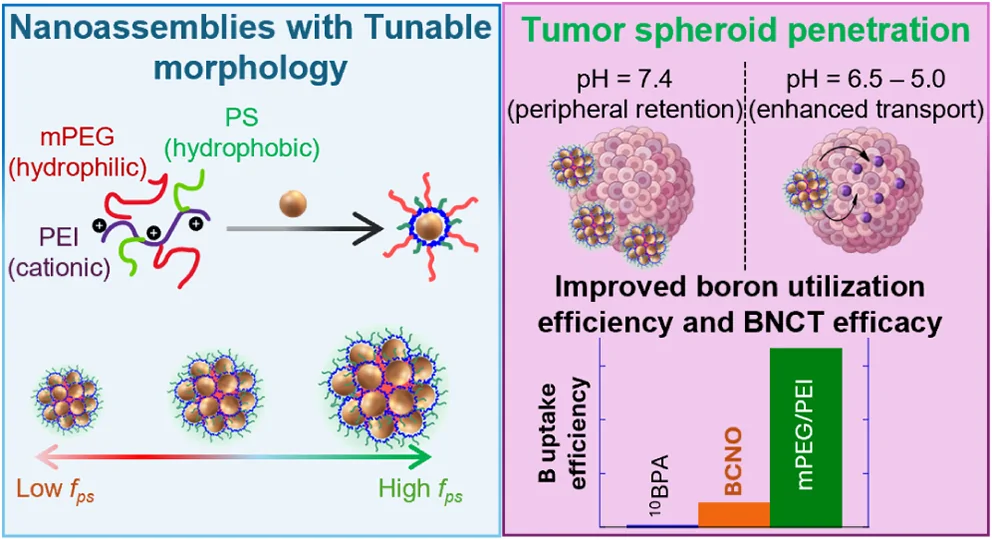

## 1 Introduction

Pancreatic cancer remains one of the most lethal malignancies, characterized by late diagnosis, rapid metastasis,^1,2^ and poor responsiveness to conventional therapies such as chemotherapy and radiotherapy.^3–5^ The dense stromal architecture,^6^ hypoxic microenvironment,^7^ and anatomical location of pancreatic tumors collectively hinder drug penetration and elevate the risk of damage to surrounding healthy tissues during treatment.^8^ These challenges underscore the urgent need for innovative therapeutic strategies with enhanced selectivity and tumor targeting capabilities.

Boron Neutron Capture Therapy (BNCT) is an emerging binary radiotherapeutic approach that relies on the selective delivery of boron-10 (^10^B) to tumor cells, followed by neutron irradiation. The neutron capture reaction generates high linear energy transfer (LET) α-particles and lithium nuclei, which destroy cancer cells with subcellular precision while sparing adjacent healthy tissues.^9–11^ BNCT holds several advantages over conventional radiotherapy, including efficacy in hypoxic tumors,^9^ circumvention of drug resistance mechanisms, and the potential to eradicate micrometastases.^9,12,13^

Despite its promise, the clinical advancement of BNCT is limited by the availability of effective boron delivery agents that can selectively accumulate in tumor tissues at therapeutically relevant concentrations. To achieve therapeutic efficacy, boron agents must meet strict criteria: they should accumulate at least 1 × 10^9 10^B per cell or 20 μg/g of ^10^B within tumor tissue and achieve a tumor-to-normal cell (T/N) or tumor-to-blood (T/B) ratio of at least 3.^14–18^ Current clinical agents, such as boronophenylalanine (BPA) and sodium borocaptate (BSH), suffer from low tumor selectivity and suboptimal intracellular delivery.^19–24^ To address these limitations, nanoparticle-based boron carriers have been explored, leveraging their high boron payload, tunable size, and surface modifiability.^25–28^ According to the enhanced permeability and retention (EPR) effect, nanoparticles sized between 50 and 200 nm can passively accumulate in tumor tissue via leaky vasculature and poor lymphatic drainage.^29–32^

Among the emerging candidates, boron carbon oxynitride (BCNO) nanoparticles have gained attention as a next-generation boron agent due to their intrinsically high boron content (up to ∼40%),^9,33,34^ favorable biocompatibility,^35^ and photoluminescence properties that enable real-time fluorescence-based imaging and tracking.^36^ However, pristine BCNO particles require surface modification to improve their colloidal stability, dispersion, and tumor-targeting capability.

To further enhance precision and delivery efficiency, recent strategies have focused on stimuli-responsive polymeric nanocarriers that release their payload in response to specific tumor-associated cues.^37–39^ In particular, amphiphilic block copolymers have emerged as powerful tools for constructing self-assembled nanostructures because of their inherent ability to form complex supramolecular architectures, enhance colloidal stability, and provide modular platforms for surface engineering. Moreover, incorporating acid-labile linkages enables pH-triggered disassembly in the acidic tumor microenvironment or within endosomal/lysosomal compartments, facilitating intracellular drug release and deeper tumor penetration.^40–42^

In this study, we designed a pH-responsive nanoassembly system by coating boron carbon oxynitride (BCNO) nanoparticles with a stimuli-responsive amphiphilic block copolymer, methoxy polyethylene glycol/poly­(styrene)-*block*-poly­(ethylenimine) (mPEG/PS-*b*-PEI). The nanostructure was engineered to combine long circulation (via PEGylation), structural integrity (via hydrophobic PS), and pH-responsive disassembly (via Schiff base linkages between PEI and aldehyde-terminated blocks). To evaluate the performance of this nanocarrier under conditions that better mimic in vivo tumor physiology, we employed a three-dimensional (3D) tumor spheroid model. Compared to traditional two-dimensional (2D) cell cultures, 3D spheroids more accurately replicate key features of solid tumors——including cell–cell interactions, oxygen and nutrient gradients, and extracellular matrix density——that strongly influence nanoparticle penetration and therapeutic efficacy.^43^ This model serves as a robust platform to assess the delivery efficiency and therapeutic potential of the developed nanoassemblies in a physiologically relevant context.

Through the synergistic combination of passive targeting, stimuli-responsive disassembly, and self-assembly, the mPEG/PS-*b*-PEI@BCNO nanoassemblies are expected to enhance intratumoral boron accumulation and penetration, offering a promising strategy for effective BNCT against refractory malignancies such as pancreatic cancer.

## 2 Materials and Instruments

### 2.1 Chemicals and Instruments

All chemicals and reagents used in this study were obtained from commercial sources and used as received without further purification. Boric acid (H_3_BO_3_, 99.5%), hexamethylenetetramine (C_6_H_12_N_4_, 99+%), guanidine hydrochloride (CH_5_N_3_ HCl, 99.5%, without anticaking agent), *N*,*N*’-dicyclohexylcarbodiimide (C_13_H_22_N_2_,DCC, 99%), branched polyethylenimine (PEI, M.W. 1,800, 99%), 2,2’-bipyridine (99+%), styrene (99%, stabilized with 10–15 ppm 4-*tert*-butylcatechol, DAPI (4’,6diamino-2-,phenylindole, dihydrochloride), FAST Dil solid; Dil Δ ^9,12^-C_18_(3), and CBS (1,1’-Dilinoleyl-3,3,3′,3′-tetramethylindocarbocyanine, 4-chlorobenzenesulfonate) were purchased from Thermo Fisher Scientific; 4-dimethylaminopyridine (DMAP, C_7_H_10_N_2_, 98%) and 4-formylbenzoic acid (C_8_H_6_O_3_) were purchased from Matrix Scientific. Dichloromethane (DCM, CH_2_Cl_2_) and anhydrous methanol were sourced from Avantor, and isopropanol (≥99.5%) was purchased from J.T. Baker. Poly­(ethylene glycol) methyl ether (mPEG, *M*_n_ ∼5,000), 2-hydroxyethyl 2-bromoisobutyrate (95%), aluminum oxide, nitric acid (HNO_3_, 70%, ACS reagent), pluronic F-127, and triton X-100 were purchased from Sigma-Aldrich. Copper­(I) bromide (CuBr, 98%) was obtained from Alfa Aesar. Diethyl ether and tetrahydrofuran (containing BHT as stabilizer) were provided by Duksan Pure Chemicals Co., Ltd. The CellTiter 96 Aqueous One Solution Reagent (MTS assay) was purchased from Promega Corporation. RapiClear 1.49 was acquired from SUNJin Lab. L-(4^10^boronophenyl) alanine injection (^10^BPA, 25 mg/mL) was supplied by Taiwan Biotech through the NTHU-BNCT Center.

#### 2.1.1 Experimental Methods

For *in vitro* experiments, UN-KC-6141 pancreatic cancer cells were kindly provided by the research laboratory of Prof. Fang-Hsin Chan’s. Cells were cultured in Dulbecco’s Modified Eagle Medium (DMEM) supplemented with 10% fetal bovine serum (FBS) and 1% penicillin-streptomycin (Pen/Strep), and maintained at 37 °C in a humidified incubator with 5% CO_2_. Cells were detached using with 0.25% trypsin EDTA for subculturing.

Polymer structures were characterized by ^1^H nuclear magnetic resonance (NMR) spectroscopy using a 700 MHz spectrometer (VARIAN VNMRS-700), with chemical shifts referenced to the tetramethylsilane (TMS) standard. Fourier-transform infrared (FTIR) spectroscopy was performed using a Bruker, Vertex 80v spectrometer, with polymer samples dissolved in anhydrous DCM. Thermal decomposition profiles were recorded using thermogravimetric analysis (TGA; Mettler-Toledo, 2-HT). The morphology and size of nanocomplexes and nanoparticles were examined using a high-contrast transmission electron microscope (Hitachi, HT7700) operating at 120 keV. Briefly, BCNO nanoparticles were dispersed in deionized water by sonication to obtain a homogeneous aqueous suspension. A small aliquot of the suspension was drop-cast onto a carbon-coated copper TEM grid and allowed to dry under ambient conditions prior to analysis. For nanoscale elemental mapping, scanning transmission electron microscopy–electron energy-loss spectroscopy (STEM–EELS) was performed using a Thermo Fisher Scientific/FEI transmission electron microscope operated at 200 kV. Elemental maps were acquired using the characteristic ionization edges of boron, carbon, nitrogen, and oxygen, corresponding to the B K-edge, C K-edge, N K-edge, and O K-edge, respectively.

Hydrodynamic diameter, size distribution, and zeta potential were measured with a particle analyzer (Malvern Zetasizer ZEN3600). Boron content was quantified via inductively coupled plasma mass spectrometry (ICP-MS, Thermo Fisher Scientific iCAP TQ, Germany). Lyophilization was carried out using a freeze dryer (KINGMECH FD4.5-8P-80 °C, Taiwan). Absorbance for the MTS assay was measured using a microplate reader (Molecular Devices SpectraMax 340PC). Fluorescent imaging was carried out using laser scanning confocal microscopy (LSCM, ZEISS LSM-780, Germany) and an Olympus IX81 Confocal Laser Scanning Microscope. BNCT experiments were conducted at the Tsing Hua Open-Pool Reactor (THOR) in Hsinchu, Taiwan.

### 2.2 Preparation of BCNO Nanoparticles

The synthesis of BCNO nanoparticles was performed following the methodology outlined in our previous publication [36]. Briefly, 6.0 g (0.1 mol) of boric acid and 3.1 g (0.03 mol) of guanidine hydrochloride were dissolved in 200 mL of deionized (DI) water. The solution was heated to 90 °C until complete dissolution of the solid components. Subsequently, 0.45 g (0.0032 mol) of hexamethylenetetramine was added, and the mixture was maintained at 90 °C until the complete evaporation of water. The resulting viscous residue was dried in an oven at 80 °C overnight, yielding a white solid, which was then pulverized using a mortar and pestle. The obtained powder was subjected to calcination in a tube furnace at 900 °C for 1 h with a heating rate of 5 °C/min under ambient atmospheric conditions. The calcined product, exhibiting a brownish hue with a yield of 31.7%, was subsequently ground into a fine powder at room temperature. For TEM and DLS analyses, the BCNO powder was dispersed in DI water via probe sonication (125 W, 20 kHz) at concentrations of 0.05 and 5 mg/mL, respectively. The resulting dispersion was filtered through a 1 μm syringe filter before analysis.

### 2.3 Synthesis of Benzaldehyde-End-Functionalized mPEG (mPEG-CHO)

The benzaldehyde-end functionalization of mPEG was carried out via Steglich esterification. Briefly, 5 g (1 mmol) of mPEG-5k, 0.475 g (3 mmol) of 4-carboxylbenzaldehyde, 0.785 g (3.8 mmol) of *N*,*N*’-dicyclohexylcarbodiimide (DCC), and 0.325 g (2.6 mmol) of 4-dimethylaminopyridine (DMAP) were dissolved in 60 mL dichloromethane and stirred at 30 °C for 24 h. The resulting solution was filtered twice, and the filtrate was collected and concentrated using a rotary evaporator. The concentrated product was then dissolved in isopropanol and cooled at 4 °C for 6 h to induce crystallization. The obtained solid was filtered using cold isopropanol, collected, and subsequently recrystallized in isopropanol. The recrystallized product was dissolved in diluted hydrogen chloride, followed by liquid–liquid extraction with DCM to remove residual DMAP. The DCM layer was collected, concentrated, and precipitated in cold ether. The resulting white solid was collected, dried in an oven at 40 °C, and pulverized using a mortar and pestle. The reaction yield was 48%. The degree of functionalization and reaction completion of mPEG-CHO were assessed using ^1^H NMR spectrum and FTIR analysis. The structure of the benzaldehyde-functionalized mPEG was confirmed by ^1^H NMR spectroscopy (CDCl_3_, δ, ppm), which exhibited the following characteristic signals: 3.38 (br, methyl protons at the terminal group), 3.5–3.75 ppm (br, ethylene proton −CH_2_CH_2_O– along the mPEG backbone), 3.83–3.85 ppm (br, −CH_2_O– proton adjacent to the mPEG main chain), 4.47–4.51 ppm (br, methylene protons −COOCH_2_– adjacent to the ester end group), 7.95 ppm (br, aromatic proton C_6_H_5_CHO on benzaldehyde), 8.22 ppm (br, aromatic proton C_6_H_5_CHO on benzaldehyde), 10.1 ppm (br, aldehyde proton −CHO). The FTIR spectrum showed characteristic absorption bands at 2945 cm^–1^ (C–H stretching), 1720 cm^–1^ (C=O stretching of the ester group), and 1240 cm^–1^ (C–O–C stretching).

### 2.4 Synthesis of Hydroxyethyl-End-Terminated Polystyrene (PS) via ATRP

To synthesize polystyrene with a molecular weight of 10K via Atom Transfer Radical Polymerization (ATRP), styrene was first purified by passing it through an aluminum oxide column to remove inhibitor. CuBr was purified by sequential washing with glacial acetic acid, ethanol, and diethyl ether three times to remove impurities. Next, 100.13 mg (0.698 mmol) of CuBr and 327.06 mg (0.698 mmol) of 2–2’-bipyridine were added to a Schlenk flask, followed by alternating cycles of vacuum evacuation and nitrogen purging for 5 min each, repeated three times to remove oxygen. Meanwhile, 8 mL (6.98 mmol) of filtered styrene and 101.18 μL (0.698 mmol) of HEBIB were placed in a sample vial and purged with nitrogen for 30 min. The deoxygenated solution was injected into the Schlenk flask containing the CuBr/bipyridine complex, and the mixture was stirred until it developed a blood-red color, indicating the formation of the catalytic complex. The reaction mixture was heated to 110 °C until it solidified. The resulting polymer was dissolved in dichloromethane (DCM) and filtered through an aluminum oxide column to remove residual catalyst. The filtrate was collected, concentrated using a rotary evaporator, and subsequently precipitated in methanol. The resulting white solid was collected with 41% yield, dried in a 40 °C oven, and pulverized using a mortar and pestle. The degree of polymerization and molecular weight of the synthesized polystyrene were analyzed using ^1^H NMR spectroscopy and gel permeation chromatography (GPC). The structure of the polystyrene was confirmed by ^1^H NMR spectroscopy (CDCl_3_, δ, ppm), which exhibited the following characteristic signals: 1.3–1.7 ppm (br, methylene protons −CH_2‑_ at PS main chain), 1.7–2.1 ppm (br, methine proton −CH– at PS backbone), 6.3–6.85 ppm (br, aromatic protons on phenyl ring), and 6.86–7.21 ppm (br, aromatic protons on phenyl ring). The molecular weight and polydispersity index (*M*_w_/*M*_n_) were determined using gel permeation chromatography (GPC). The FTIR spectrum showed characteristic absorption bands at 3000–3105 cm^–1^ (aromatic C–H stretching vibrations), 2920 cm^–1^ and 2850 cm^–1^ (aliphatic C–H stretching vibrations), 1600 cm^–1^ and 1493 cm^–1^ (C=C stretching vibrations of the aromatic ring), 1452 cm^–1^ (CH_2_ bending vibrations), 1028 cm^–1^ (aromatic C–H in-plane bending), and 700 cm^–1^ and 750 cm^–1^ (aromatic C–H out-of-plane bending).

### 2.5 Synthesis of Benzaldehyde-End-Functionalized Polystyrene (PS-CHO)

The benzaldehyde-end functionalization of PS was carried out via Steglich esterification. Briefly, 2.5 g (0.25 mmol) of PS-10K, 0.475 g (3 mmol) of 4-carboxylbenzaldehyde, 0.785 g (3.8 mmol) of *N*,*N*’-dicyclohexylcarbodiimide (DCC), and 0.325 g (2.6 mmol) of 4-dimethylaminopyridine (DMAP) were dissolved in 15 mL dichloromethane and stirred at 30 °C for 24 h. The resulting solution was filtered twice, and the filtrate was collected and concentrated using a rotary evaporator. The concentrated product was then dissolved in isopropanol and cooled at 4 °C for 6 h to induce crystallization. The obtained solid was filtered using cold isopropanol, collected, and subsequently recrystallized in isopropanol. The recrystallized product was dissolved in diluted hydrogen chloride, followed by liquid–liquid extraction with DCM to remove residual DMAP. The DCM layer was collected, concentrated, and precipitated in cold ether. The resulting white solid was collected, dried in an oven at 40 °C, and pulverized using a mortar and pestle. The reaction yield was 56%. The degree of functionalization and reaction completion of PS-CHO were assessed using ^1^H NMR spectrum and FTIR analysis. The structure of the PS-CHO was confirmed by ^1^H NMR spectroscopy (CDCl_3_, δ, ppm), which exhibited the following characteristic signals: 1.3–1.7 (br, methylene protons −CH_2‑_ at PS main chain), 1.7–2.1 ppm (br, methine proton −CH– at PS backbone), 6.3–6.85 ppm (br, aromatic protons on phenyl ring), 6.86–7.21 ppm (br, aromatic protons on phenyl ring). 7.92 ppm (br, aromatic proton C_6_H_5_CHO on benzaldehyde), 8.15 ppm (br, aromatic proton C_6_H_5_CHO on benzaldehyde), and 10.1 ppm (br, aldehyde proton −CHO). The molecular weight and polydispersity index (*M*_w_/*M*_n_) were determined using gel permeation chromatography (GPC). The FTIR spectrum showed characteristic absorption bands at 3000–3105 cm^–1^ (aromatic C–H stretching vibrations), 2920 cm^–1^ and 2850 cm^–1^ (aliphatic C–H stretching vibrations), 1600 cm^–1^ and 1493 cm^–1^ (C=C stretching vibrations of the aromatic ring), 1452 cm^–1^ (CH_2_ bending vibrations), 1028 cm^–1^ (Aromatic C–H in-plane bending), 700 cm^–1^ and 750 cm^–1^ (Aromatic C–H out-of-plane bending), and 1720 cm^–1^ (C=O stretching of the ester group).

### 2.6 Synthesis of mPEG/PS-*b*-PEI Amphiphilic Block Copolymer

The synthesis of the mPEG/PS-*b*-PEI amphiphilic block copolymer was carried out using a feeding ratio of mPEG_5k_:PS_10k_:PEI_1.8k_ = 1:2:5. Initially, 50 mg (0.01 mmol) of mPEG-CHO and 200 mg (0.02 mmol) of PS-CHO were added to 5 mL of tetrahydrofuran (THF).

In a separate preparation, 90 mg (0.05 mmol) of PEI was dissolved in 5 mL of deionized (DI) water. The PEI solution was then added dropwise to the mPEG-CHO/PS-CHO solution, leading to the formation of a white suspension. To a homogeneous and transparent solution, an additional 25 mL of THF was introduced, and the reaction mixture was stirred at room temperature for 24 h to facilitate the formation of Schiff base bonds between the polymer components. The resulting solution was then lyophilized to obtain a white powder, and the successful formation of Schiff base bonds was further confirmed through NMR spectroscopy. The ^1^H NMR spectrum (CDCl_3_, δ, ppm) exhibited characteristic signals at 6.86–7.21 ppm (br, aromatic protons on phenyl ring of polystyrene), 3.50–3.75 ppm (br, ethylene proton of the −CH_2_CH_2_O– units in the mPEG backbone), 2.3–3.0 ppm (br, methylene protons of the branched PEI), and 7.97 ppm (br, imine proton −CH=N), verifying the structural integrity of the mPEG/PSb-PEI copolymer. The FTIR spectrum showed characteristic absorption bands at 3358 and 3400 cm^–1^ (N–H stretching vibrations of branched PEI), 1645 cm^–1^ (stretching vibration of imine bond C=N), 1240 cm^–1^ (C–O–C stretching vibration), 3000–3105 cm^1^ (aromatic C–H stretching vibrations), 1600 cm^–1^ and 1493 cm^–1^ (C=C stretching vibrations of the aromatic ring), and 1452 cm^–1^ (aromatic ring bending vibrations).

### 2.7 Preparation of mPEG/PS-*b*-PEI@BCNO Nanoassemblies

For the preparation of mPEG/PS-*b*-PEI@BCNO nanoassemblies, 150 mg of BCNO was dissolved in 30 mL of DI water, filtered through a 0.22 μm syringe filter, and subsequently added dropwise to the mPEG/PS-*b*-PEI solution. To investigate the self-assembly mechanism in response to variations in hydrophobicity, three different feeding ratios of the amphiphilic block copolymer mPEG/PS-*b*-PEI were prepared. In these formulations, the molar ratio of (mPEG:PS):PEI was maintained at 3:5, while the ratio between mPEG and PS was systematically adjusted to 1:2, 1:1, and 4:1, respectively. The mixture was stirred continuously until the complete evaporation of THF, promoting micelle formation. The resulting dispersion was centrifuged at 8000 rpm for 10 min, and this process was repeated three times to remove excess polymer. The final product, a gray solid, was collected and subsequently lyophilized into a gray powder. The zeta potential of the nanoassemblies was measured using DLS to evaluate their surface charge characteristics, while the morphology and size of the nanoassemblies were confirmed by TEM. The lyophilized powder was redispersed in DI water via probe sonication (125 W, 20 kHz) at concentrations of 5 and 0.05 mg/mL, respectively.

### 2.8 mPEG/PS-*b*-PEI@BCNO Nanoassemblies at Different pH Values

The influence of pH on the self-assembly behavior of mPEG/PS-*b*-PEI@BCNO nanoassemblies was examined by adjusting the pH using 1 M HCl solution. To prepare a pH 5 buffer, 2 × 10^–6^ mL of 1 M HCl was added into 200 mL of DI water. Subsequently, 3.16 × 10^–3^ of the pH 5 solution was diluted with 100 mL of DI water to obtain a solution with a final pH of 6.5. The synthesized mPEG/PS-*b*-PEI@BCNO nanoassemblies (20 mg) were dispersed in 4 mL of pH 6.5 and pH 5 solutions, respectively. These dispersions were incubated for a predetermined period to potential cleavage of the Schiff base bonds under acidic conditions. After incubation, the samples were centrifuged at 8000 rpm for 10 min to remove the acid solution. The supernatant was discarded, and the resulting pellets were redispersed in 5 mL of DI water to neutralize the samples. For TEM analysis, the samples were subjected to probe sonicated (125 W, 20 kHz) for 6 s, filtered through a 1 μm syringe filter, and then deposited onto carbon-coated copper grids. The cleavage of the Schiff base linkages was further confirmed by ^1^H NMR spectroscopy, which revealed a characteristic broad signal at 10.1 ppm corresponding to the aldehyde proton (−CHO).

### 2.9 Cytotoxicity of mPEG/PS-*b*-PEI@BCNO in UN-KC-6141 Pancreatic Cancer Cells

2.9.1

The cytotoxicity of the synthesized BCNO nanoassemblies was assessed using the CellTiter 96 Aqueous One Solution Cell Proliferation Assay (MTS assay). UN-KC6141 pancreatic cancer cells were selected as the model cell line for this study. Cells were seeded at a density of 7.0 × 10^3^ cells per well in a 96-well plate and incubated for 24 h under standard culture conditions. Following incubation, the cells were washed twice with phosphate-buffered saline (PBS). BCNO nanoparticles, mPEG/PSb-PEI@BCNO nanoassemblies, and boronophenylalanine (BPA) were dispersed in culture medium at concentrations of 1000, 500, 250, 125, 62.5, 31.25, and 15.6 μg/mL. Cells were treated with these formulations at the indicated concentrations for 24, 48, and 72 h. At each time point, 10 μL of MTS reagent was added to each well, followed by a 2-h incubation period (*n* = 3). The absorbance was measured at 490 nm using a microplate reader. Cell viability was calculated based on the absorbance reading, with untreated cells serving as the negative control (set as 100% viability).

$$Cell\;viability=\frac{OD_{sample}-OD_{blank}}{OD_{control}-OD_{blank}}\times 100\%$$

### 2.10 Quantification of Cellular Uptake of mPEG/PS-*b*-PEI@BCNO

The cellular uptake of nanoparticles was quantitatively analyzed using inductively coupled plasma mass spectrometry (ICP-MS). UN-KC-6141 cells were seeded in a 6-well plate at a density of 3.0 × 10^5^ cells per well and incubated for 24 h. The cells were then treated with 31.25 μg/mL of BPA, BCNO nanoparticles, and mPEG/PS-*b*-PEI@BCNO nanoassemblies. After incubation periods of 12 and 24 h, the medium was removed, and cells were washed twice with PBS 1×, pH 7.4). Cells were subsequently harvested by trypsinization, collected by centrifugation, and digested in a mixture of 1 mL nitric acid and 10 μL of hydrofluoric acid. The intracellular boron concentration was then determined by ICP-MS (*n* = 3).

### 2.11 Evaluation of Boron Uptake in 2D Cell Culture via Confocal Microscopy

The cellular uptake of nanoparticles within 3D spheroids was qualitatively visualized using laser scanning confocal microscopy (LSCM). Cells were seeded at a density of 2.0 × 10^4^ cells per well in 24-well plates, each containing a sterilized glass coverslip, and incubated for 24 h. Cells were then treated with 31.25 μg/mL of BCNO and mPEG/PS-*b*-PEI@BCNO for 12 and 24 h. After treatment, the medium was removed, and cells were washed twice with PBS. The cells were fixed with 4% paraformaldehyde for 10 min and permeabilized with 0.1% Triton X-100 for 5 min. Subsequently, the cell membranes were stained with FAST-Dil (1:5000 dilution), and nuclei were counterstained with Hoechst 33342 (1:10,000 dilution) for 20 min. Finally, the coverslips were mounted on glass slides for fluorescence imaging using LSCM.

### 2.12 In Vitro BNCT Treatment

UN-KC-6141 pancreatic cancer cells were seeded in 96-well plates at a density of 7.0 × 10^3^ cells per well and incubated for 24 h. The cells were then treated with 31.25 μg/mL of BNCO, mPEG/PS-*b*-PEI@BNCO and BPA for 12 and 24 h, respectively. Following treatment, the culture medium was removed, and the cells were washed twice with PBS to eliminate uninternalized boron compounds. The 96-well plate was subsequently mounted onto an acrylic holder and subjected to BNCT at the Tsing Hua Open Pool Reactor (THOR), operating at a power of 1.2 MW, with a thermal neutron flux of 1 × 10^9^ neutrons/cm^2^s for a duration of 30 min. After irradiation, the cells were further incubated for 24 and 48 h to assess post-treatment viability using the MTS assay (*n* = 6).

### 2.13 Establishment of an In Vitro 3D Spheroid Model of UN-KC-6141 Pancreatic Cells

To establish a three-dimensional (3D) spheroid model, nontreated round-bottom 96-well plates were initially coated with 200 μL of 0.7% Pluronic F-127 solution per well and incubated at 37 °C for 1 h to prevent cell adhesion. Following incubation, the coating solution was removed, and the wells were rinsed with PBS. After the PBS was discarded, UN-KC-6141 pancreatic cancer cells were seeded at a density of 10,000 cells per well. After 4 days of incubation under standard culture conditions, uniform spheroids with an average diameter of approximately 400 μm were formed.

### 2.14 Penetration of Nanoparticles in 3D Tumor Spheroids

The penetration of nanoparticles within 3D spheroids was qualitatively visualized using laser scanning confocal microscopy (LSCM). The culture medium of mature spheroids was replaced with fresh medium containing 31.25 μg/mL of either BCNO or mPEG/PS-*b*-PEI@BCNO at pH 7.4 and pH 6.5. The spheroids were incubated for 6, 12, 24, and 72 h under standard culture conditions. Following treatment, the medium was removed, and spheroids were washed twice with PBS. The spheroids were then fixed with 4% paraformaldehyde for 10 min and permeabilized with 0.1% Triton X-100 for 15 min. Optical clearing was performed using RapiClear 1.49 to enhance transparency. Nuclei were stained with Hoechst 33342 (1:10,000 dilution) for 1 h. Finally, the spheroids were transferred to imaging chambers and mounted on glass slides for fluorescence observation by LSCM.

### 2.15 Boron Internalization in 3D Tumor Spheroids

The cellular uptake of nanoparticles within 3D spheroids was quantitatively assessed by inductively coupled plasma mass spectrometry (ICP-MS). The spheroids were treated under two experimental conditions: (1) a fixed concentration of 31.25 μg/mL of BPA, BCNO, or mPEG/PS-*b*-PEI@BCNO; and (2) an equivalent fixed ^10^B concentration using 31.25 μg/mL of mPEG/PS-*b*-PEI@BCNO, 2.5 μg/mL of BCNO, and 3.26 μg/mL of BPA. Treatments with BCNO and mPEG/PS-*b*-PEI@BCNO were performed at both pH 7.4 and pH 6.5. All treatments were conducted for 72 h under standard culture conditions. Following incubation, the medium was removed, and the spheroids were washed twice with PBS. A total of 15 spheroids per group were collected, centrifuged, and digested in a mixture of 1 mL nitric acid and 10 μL of hydrofluoric acid. The intracellular boron content was subsequently determined using ICP-MS (*n* = 3).

### 2.16 In Vitro 3D Spheroid BNCT Treatment

UN-KC-6141 spheroids were treated under two experimental conditions: (1) a fixed concentration of 31.25 μg/mL of BPA, BCNO, or mPEG/PS-*b*-PEI@BCNO; and (2) an equivalent fixed ^10^B concentration using 31.25 μg/mL of mPEG/PS-*b*-PEI@BCNO, 2.5 μg/mL of BCNO, and 3.26 μg/mL of BPA. Treatments involving BCNO and mPEG/PSb-PEI@BCNO were conducted at both pH 7.4 and pH 6.5. All treatment groups were preincubated for 72 h under standard culture conditions prior to BNCT. Following incubation, the culture medium was removed, and spheroids were washed twice with PBS to eliminate uninternalized boron compounds. The 96-well plate was secured onto an acrylic holder and subjected to BNCT at the Tsing Hua Open Pool Reactor (THOR), operating at a power of 1.2 MW with a thermal neutron flux of 1 × 10^9^ neutrons/cm^2^s for 30 min. Postirradiation, spheroids were incubated for an additional 24 h to evaluate cell viability using the MTS assay (*n* = 5).

### 2.17 Statistical Analysis

Statistical analysis was performed using the paired comparison tool in Origin 2025 (OriginLab), followed by Tukey’s post hoc test. Statistical significance was denoted as *P ≤ 0.05, **P ≤ 0.01, ***P ≤ 0.001.

## 3 Results and Discussion

### 3.1 Macromolecular Design Strategy and Synthesis of mPEG/PS-*b*-PEI

The PEG/PS-*b*-PEI@BCNO nanoassemblies were designed as a macromolecular system in which boron delivery efficiency is governed by polymer architecture rather than boron loading alone. The amphiphilic block copolymer integrates three orthogonal design elements: a PEG corona to stabilize colloidal dispersion and modulate interfacial interactions, a hydrophobic PS segment to promote nanoassembly integrity during self-assembly, and a polycationic PEI domain that enables electrostatic complexation with negatively charged BCNO nanoparticles. Importantly, dynamic Schiff base linkages were incorporated between polymer segments to introduce environmental responsiveness without compromising assembly fidelity. This modular architecture allows the nanoassemblies to maintain stability under physiological conditions while enabling adaptive structural reorganization in tumor-relevant environments, thereby maximizing boron utilization per unit mass rather than the absolute boron payload. The synthetic route of the amphiphilic mPEG/PS-*b*-PEI block copolymer and the preparation of PEG/PS-*b*-PEI@BCNO nanoassemblies are illustrated in Scheme 1.

**Scheme 1:**
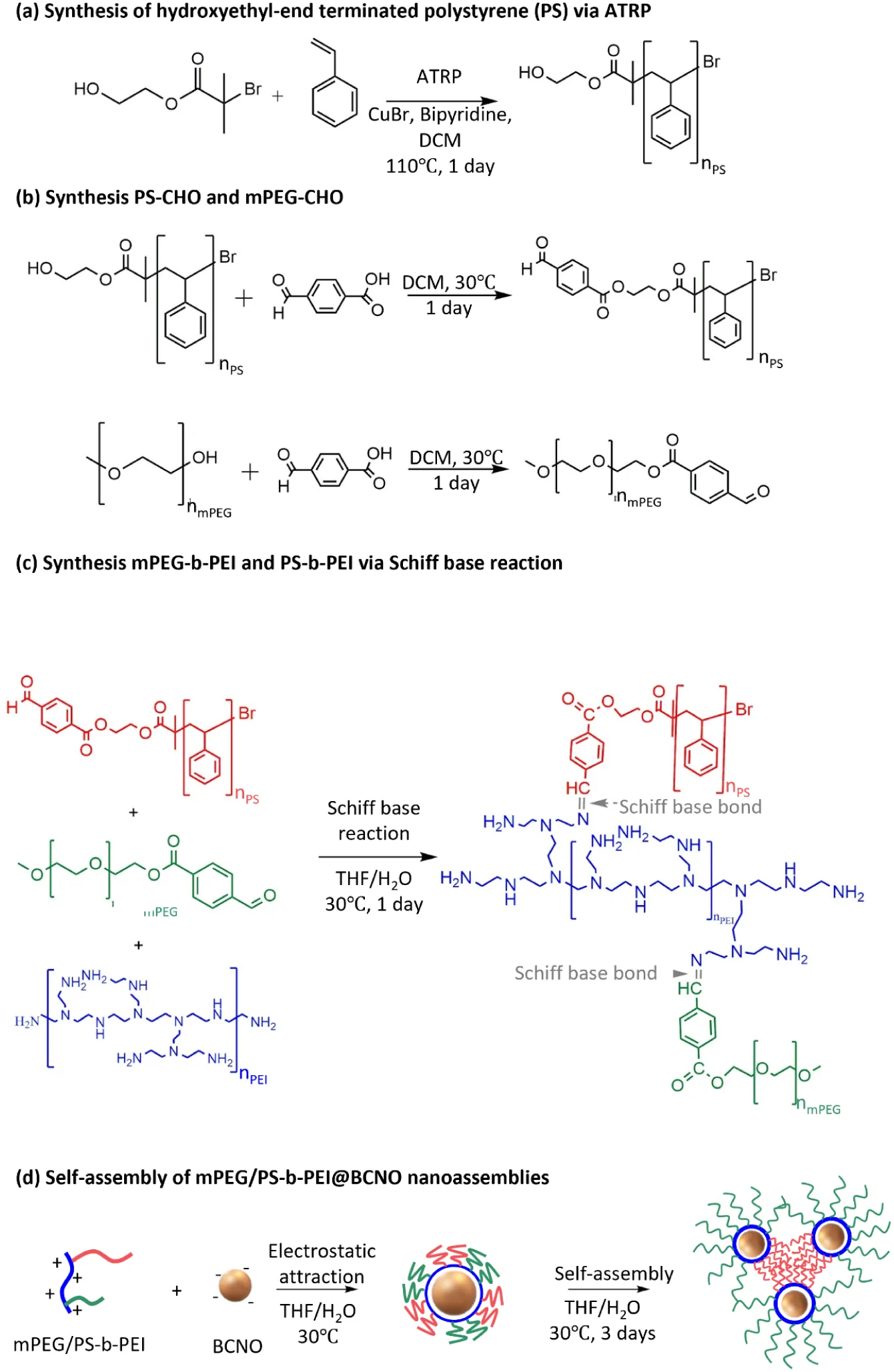
Schematic Illustration of the Synthetic Procedures: (a) Synthesis of Hydroxyethyl-Terminated Polystyrene (PS); (b) Functionalization of PS and mPEG with 4-Carboxybenzaldehyde to Obtain Aldehyde-Terminated PS–CHO and mPEG–CHO; (c) Formation of the Amphiphilic Block Copolymer mPEG/PS-*b*-PEI via Schiff Base Reaction with Hyperbranched PEI; and (d) Self-Assembly of mPEG/PS-*b*-PEI@BCNO Nanoassemblies in a THF/Water Mixture

The synthesis began with the preparation of hydroxyethyl-terminated polystyrene (PS) via atom transfer radical polymerization (ATRP) (Scheme 1a). Subsequently, both PS and methoxy polyethylene glycol (mPEG) were functionalized with 4-carboxybenzaldehyde through a standard DCC coupling reaction to introduce aldehyde functionalities at their termini, yielding PS–CHO and mPEG–CHO, respectively (Scheme 1b). These aldehyde-terminated polymers were then conjugated to hyperbranched polyethylenimine (PEI) via a nucleophilic addition reaction between the aldehyde groups and the primary amines of PEI, forming hemiaminal intermediates.

These intermediates were subsequently dehydrated to generate dynamic Schiff base (imine) linkages, resulting in the formation of the amphiphilic block copolymer mPEG/PS-*b*-PEI (Scheme 1c). Due to the protonation of amino groups in PEI under aqueous conditions, the resulting mPEG/PS-*b*-PEI copolymer carries a positive surface charge in deionized (DI) water. In contrast, the BCNO nanoparticles exhibit a negative surface charge due to the presence of surface hydroxyl groups. Electrostatic attraction between the oppositely charged components facilitates the coating of BCNO by the amphiphilic block copolymer. Finally, the mPEG/PS-*b*-PEI@BCNO nanoassemblies were formed via self-assembly in a THF/water mixture, driven by the amphiphilic nature of the copolymer (Scheme 1d).

### 3.2 Characterization of BCNO Nanoparticles

The synthesis and characterization of BCNO nanoparticles were previously reported in our earlier study.^36^ To confirm the successful formation of the BCNO nanoparticles, multiple analytical techniques were employed, including photoluminescence (PL) spectroscopy, X-ray diffraction (XRD), X-ray photoelectron spectroscopy (XPS), transmission electron microscopy (TEM), and zeta potential analysis. The as-synthesized BCNO nanoparticles, obtained after calcination at 900 °C for 1 h, exhibited visible photoluminescence in aqueous suspension (Figure S1a). Under excitation at 365 nm, the PL emission spectrum displayed a broad peak centered around 450 nm, indicating the intrinsic self-luminescent property of the BCNO nanoparticles (Figure S1b). This property offers a significant advantage for noninvasive tracking of drug distribution in biological systems.

TEM analysis revealed that the BCNO nanoparticles exhibited a uniform spherical morphology with an average diameter of 7.63 ± 1.05 nm (*n* = 30) (Figure 1a). High-resolution TEM revealed discernible lattice fringes, and Fast Fourier Transform (FFT) analysis of the HRTEM image (Figure 1b–d) yielded a lattice spacing of approximately 0.338 nm, consistent with the (002) plane of turbostratic boron nitride (t-BN), indicative of disordered layer stacking. XRD analysis further corroborated this structural assignment, displaying broad diffraction peaks centered around 26.6° and 43.1° (2θ), corresponding to the (002) and (100) planes, respectively (Figure 1e). These features are characteristic of t-BN and are in good agreement with our previous reports.^36^

**Figure 1:**
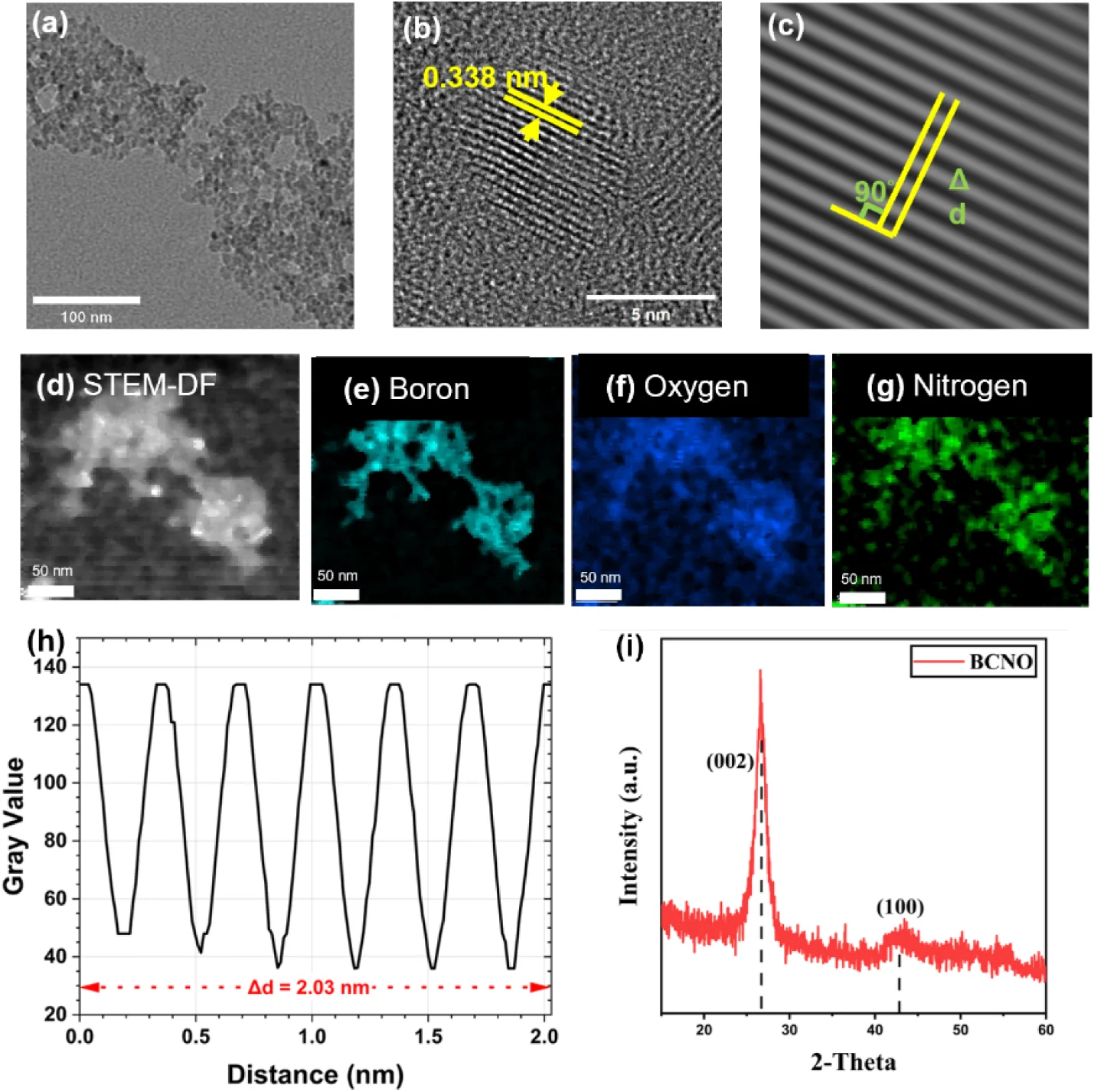
Characteristics of BCNO nanoparticles: (a) Transmission electron microscopy (TEM) image showing the morphology of BCNO nanoparticles. (b) High-resolution TEM (HR-TEM) image revealing the lattice spacing of the BCNO nanoparticles. (c) Inverse FFT imaging obtained from the HRTEM image. (d) STEM dark-dark field image of BCNO and the corresponding electron energy-loss spectroscopy (EELS) elemental mapping images of (e) boron, (f) oxygen, and (g) nitrogen. (h) Plot profile diagram of the inverse FFT image. (i) X-ray diffraction (XRD) pattern of the synthesized BCNO nanoparticles.

XPS surface analysis confirmed a boron content of approximately 37.3% (Table S1), validating the incorporation of boron within the nanoparticle matrix. Additionally, zeta potential measurements indicated a surface charge of −21.7 mV (Figure S1c), indicating that the negatively charged BCNO nanoparticles could form BCNO nanoassemblies with the positively charged mPEG/PS-*b*-PEI amphiphilic block copolymer through electrostatic complexation.^44–47^

### 3.3 Synthesis of Hydroxyethyl-End-Terminated Polystyrene (PS) via ATRP

Hydroxyethyl-terminated polystyrene was synthesized via atom-transfer radical polymerization (ATRP) using a monomer-to-initiator ratio of 100:1. The resulting polymer exhibited a high monomer conversion and controlled molecular weight characteristics. As shown in the ^1^H NMR spectrum^48^ (Figure 2a), the characteristic aromatic and aliphatic backbone signals of polystyrene were clearly observed, while residual vinyl protons from unreacted styrene were minimal (Figure S2a), corresponding to a monomer conversion of 93.36% as calculated from NMR integration (see Supporting Information).

**Figure 2:**
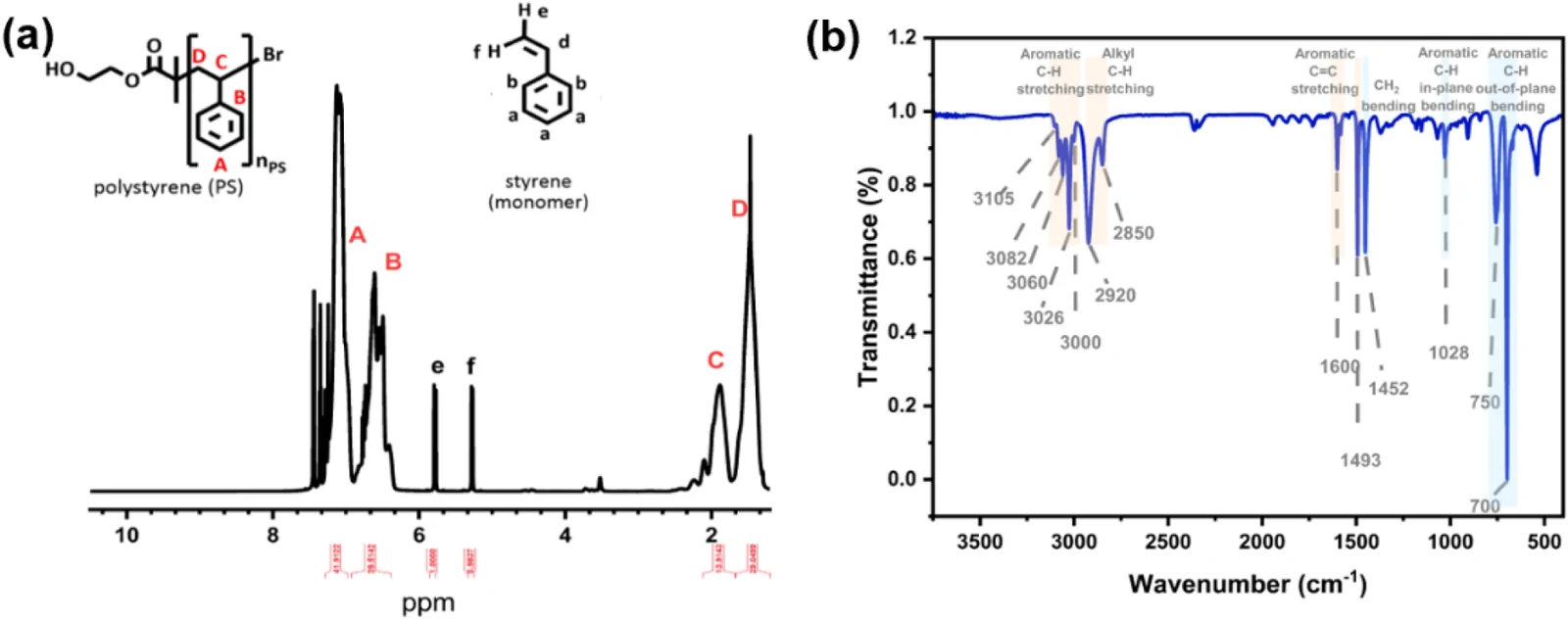
(a) ^1^H NMR spectrum of crude polystyrene. (b) FTIR spectrum of the synthesized polystyrene, confirming its characteristic functional groups.

The degree of polymerization (DP) was determined by NMR to be 93, yielding a number-average molecular weight (Mn NMR) of approximately 9700 g/mol. Gel permeation chromatography (GPC) further confirms the controlled nature of the polymerization, affording an Mn GPC of 10,900 g/mol with a narrow polydispersity index of 1.08 (Figure S2b and Table S2). The close agreement between the NMR- and GPC-derived molecular weights indicates efficient initiation and minimal termination during polymer growth.

FTIR analysis (Figure 2b) displayed absorption bands characteristic of polystyrene,^49,50^ further confirming successful polymer formation. Detailed peak assignments and spectral analysis are provided in the Supporting Information.

### 3.4 Characterization of Benzaldehyde-End-Functionalized mPEG and PS (mPEG-CHO and PS-CHO)

Aldehyde-terminated mPEG (mPEG-CHO) and polystyrene (PS-CHO) were achieved via DCC-mediated Steglich esterification and confirmed by ^1^H NMR and FTIR spectroscopy. In the ^1^H NMR spectrum of mPEG-CHO (Figure 3a), a distinct aldehyde proton signal was observed at ∼10.1 ppm, accompanied by aromatic protons of the 4-formylbenzoate end group at 8.0–8.2 ppm, confirming successful end-group modification. The aldehyde-to-polymer integration, normalized to the PEG backbone resonance at ∼3.6 ppm, gave an approximate functionalization efficiency of 97%. FTIR analysis further supported aldehyde incorporation, with the emergence of a characteristic C=O stretching band at ∼1720 cm^–1^ (Figure 3b).

**Figure 3:**
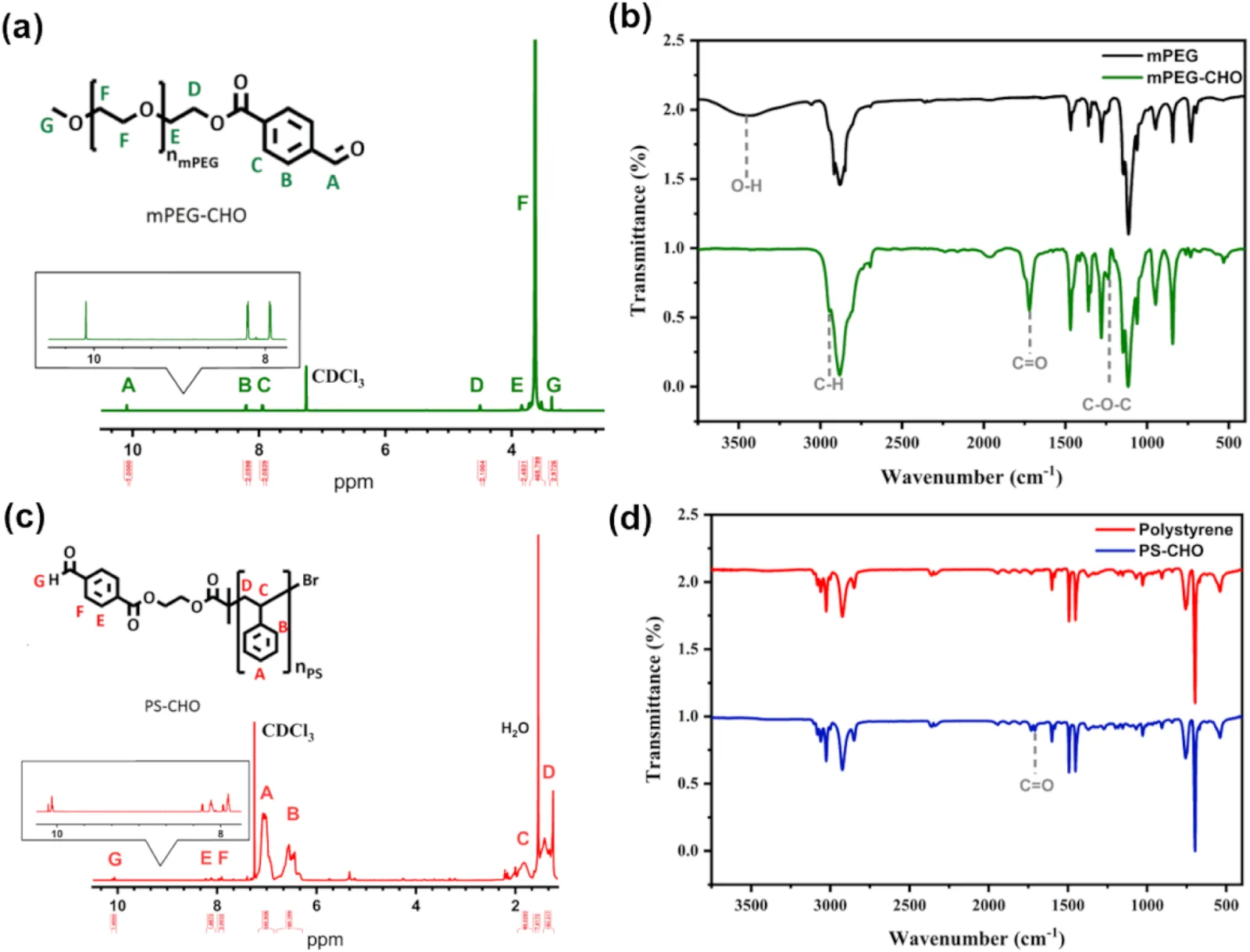
(a) ^1^H NMR spectrum of the mPEG-CHO recorded in CDCl_3_ at ambient temperature, confirming the successful functionalization of mPEG with benzaldehyde groups. (b) FTIR spectrum of mPEG-CHO, confirming the successful functionalization of mPEG with benzaldehyde moieties. (c) ^1^H NMR spectrum of PS-CHO. (d) FTIR spectrum of PS-CHO, confirming the successful functionalization of polystyrene with benzaldehyde moieties.

Similarly, PS-CHO exhibited a clear aldehyde proton signal at ∼10.1 ppm in the ^1^H NMR spectrum, together with aromatic resonances from both the benzaldehyde unit and the polystyrene backbone (Figure 3c). The aldehyde-to-polymer integration, normalized to the PS aromatic backbone using the degree of polymerization determined for the PS, gave an approximate functionalization efficiency of ∼86%. The corresponding FTIR spectrum displayed a distinct carbonyl stretching band at ∼1720 cm^–1^, absent in unmodified PS, confirming successful aldehyde installation (Figure 3d). GPC analysis showed that molecular weight and dispersity were well preserved after functionalization (Figure S2c), indicating minimal chain scission or coupling during the esterification process. Collectively, these results confirm efficient and controlled aldehyde end-group functionalization of both polymer segments, providing reactive handles for subsequent Schiff-base conjugation with polyethylenimine (PEI). This strategy enables the construction of amphiphilic block copolymers with precisely defined hydrophilic, hydrophobic, and cationic domains required for nanoassembly and boron delivery applications.

### 3.5 Synthesis and Composition Analysis of mPEG/PS-*b*-PEI Amphiphilic Block Copolymer

The amphiphilic mPEG/PS-*b*-PEI copolymer was synthesized via a Schiff base condensation reaction between benzaldehyde-terminated mPEG and PS, and the primary amine groups of hyperbranched polyethylenimine.^51^ Using a representative feeding ratio of mPEG (5 000 g/mol), PS (10 000 g/mol), and PEI (1 800 g/mol) of 1:2:5, the reaction proceeded efficiently under mild conditions, yielding the targeted multifunctional copolymer.

Successful copolymer formation was confirmed by ^1^H NMR spectroscopy (Figure 4a). Characteristic resonances corresponding to the mPEG backbone (∼3.6 ppm), aromatic protons of the PS segments (∼6.5–7.0 ppm), and methylene protons of PEI (2.3–3.0 ppm) were clearly observed.^52^ Notably, the disappearance of the aldehyde proton signal at ∼10.1 ppm indicates near-complete consumption of aldehyde end groups and formation of Schiff-base linkages.

**Figure 4:**
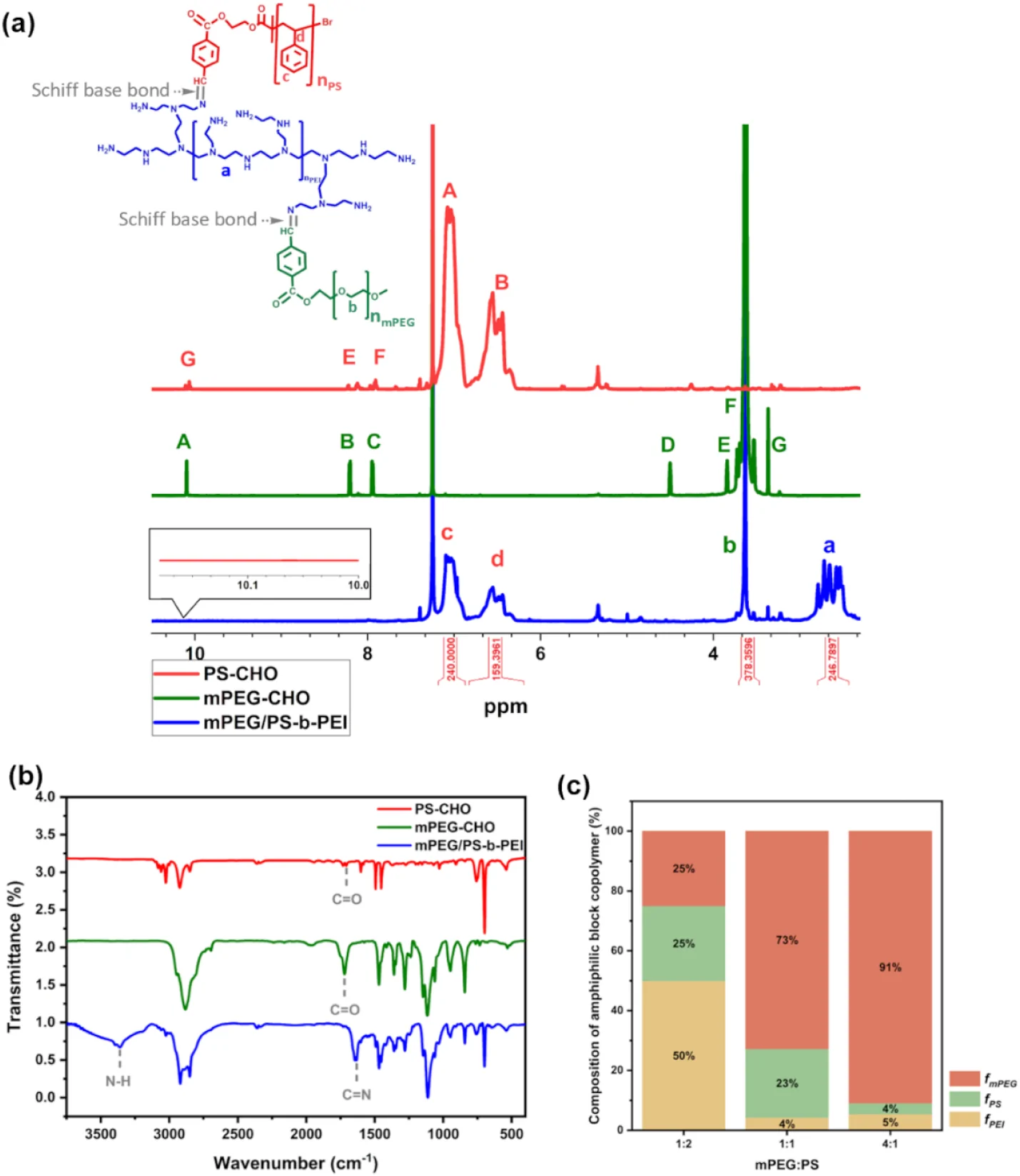
(a) ^1^H NMR spectrum of mPEG/PS-*b*-PEI confirming the formation of Schiff base linkages and elucidating the copolymer composition. (b) FTIR spectrum of the mPEG/PS-*b*-PEI amphiphilic block copolymer, confirming the successful formation of the copolymer through characteristic functional group analysis. (c) Molar composition of mPEG/PS-*b*-PEI synthesized with varying feeding ratios, as determined by ^1^H NMR spectroscopy.

FTIR spectroscopy further corroborated successful conjugation (Figure 4b). The carbonyl stretching band of the aldehyde group at 1720 cm^–1^, present in both PS-CHO and mPEG-CHO, was absent in the copolymer spectrum, while a weak band at ∼1645 cm^–1^ corresponding to imine (C=N) stretching emerged, consistent with Schiff-base formation. These spectroscopic results confirm effective covalent coupling between the mPEG/PS segments and PEI.

The molar composition of the resulting copolymer was estimated from ^1^H NMR integration of the respective polymer segments,^53^ yielding an approximate ratio of mPEG:PS:PEI ≈1:1:2. To examine the influence of polymer composition on self-assembly behavior, additional copolymers with varied mPEG and PS feeding ratios were prepared. The corresponding segmental molar fractions, determined by NMR analysis, are summarized in Figure 4c, providing a quantitative framework for correlating copolymer composition with nanoassembly morphology and properties, as discussed in the following section. Detailed compositional calculations are provided in the Supporting Information.

### 3.6 Characteristics of mPEG/PS-*b*-PEI@BCNO Nanoassemblies

In this study, the mPEG/PS-*b*-PEI amphiphilic block copolymer was employed to modify the surface properties, morphology, and size of BCNO nanoparticles through electrostatic complexation and solvent-driven self-assembly. The nanoassemblies were prepared by introducing an aqueous suspension of BCNO into a tetrahydrofuran (THF) solution of the amphiphilic block copolymer, followed by the gradual evaporation of the THF. Under these conditions, the hydrophobic polystyrene (PS) segments collapsed in the aqueous environment, driving the formation of polymer-tethered BCNO nanoassemblies through microphase segregation of the amphiphilic copolymer.^54^

To investigate the influence of polymer composition on the resulting nanostructures, the relative fractions of hydrophobic PS and hydrophilic mPEG were systematically varied. TEM imaging revealed distinct morphological transitions in the self-assembled nanostructures as the *f*_PS_ decreased from 0.25 to 0.23 (Figure 5a–d). At higher hydrophobic content (*f*_PS_ *=* 0.25), uniform spherical nanoassemblies with an average diameter of 61.0 ± 10.3 nm were obtained. Reducing *f*_PS_ to 0.23 produced smaller clustered structures (diameter: 25.4 ± 7.2 nm), while further reduction to 0.04 yielded predominantly discrete nanoparticles with an average diameter of 18.2 ± 5.7 nm. This morphological and dimensional transition is attributed to the reduced hydrophobic interactions, which lowered the packing number of nanoparticles within each assembly. Concurrently, the increase in *f*_mPEG_ contributed to enhanced steric repulsion at the assembly interface, further promoting the formation of isolated nanoparticles (Figure 5f).

**Figure 5:**
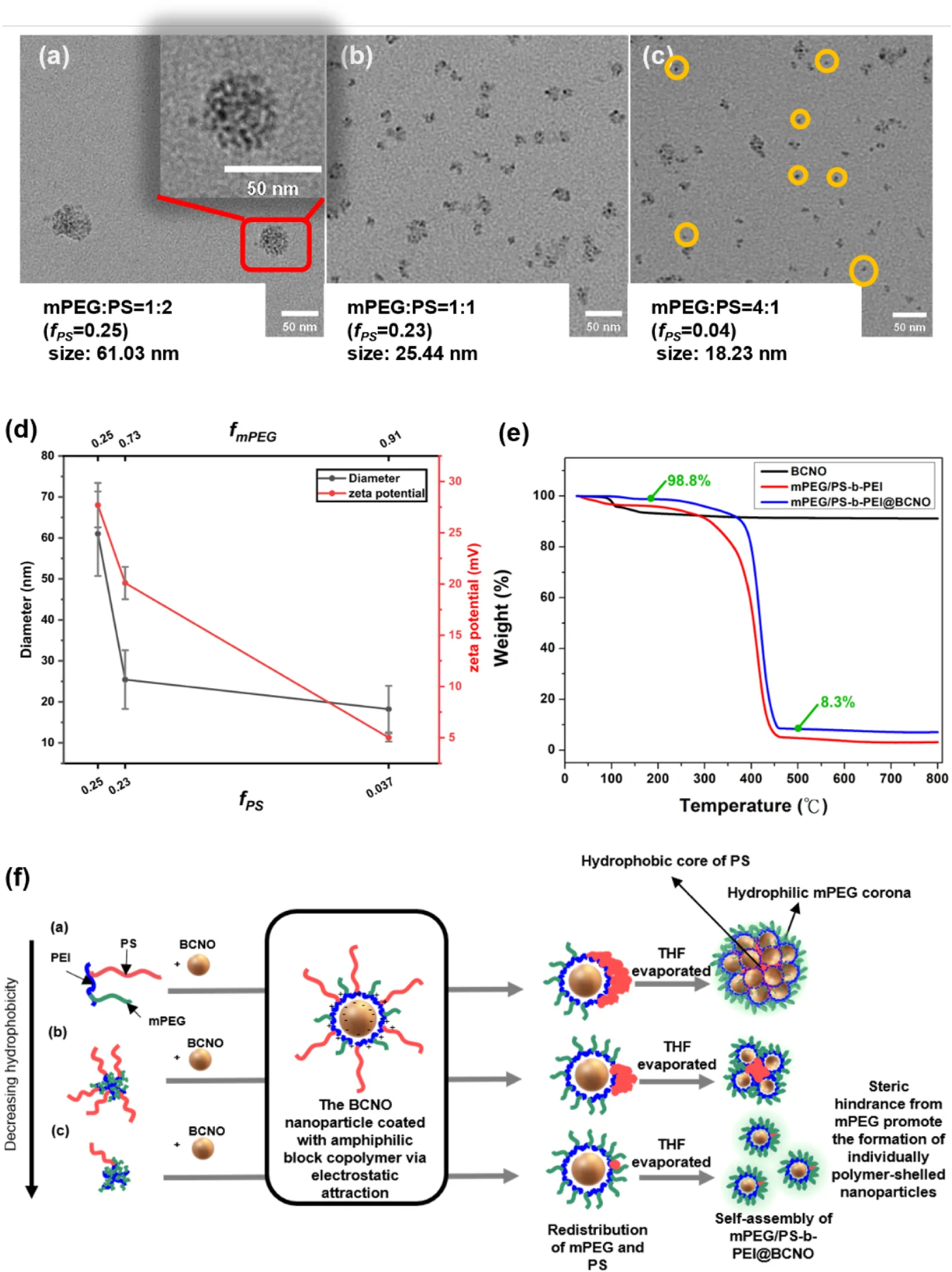
TEM images of mPEG/PS-*b*-PEI@BCNO nanoassemblies prepared with varying mPEG:PS feeding ratios: (a) 1:2, (b) 1:1, and (c) 4:1. Orange circles indicate the presence of a single nanoparticle. Scale bar: 50 nm. (d) Diameter and zeta potential measurements of mPEG/PS-*b*-PEI@BCNO nanoassemblies, corresponding to their respective molar compositions. (e) TGA curves of bare BCNO, mPEG/PS-*b*-PEI, and mPEG/PS-*b*-PEI@BCNO, illustrating the thermal stability and composition of the nanoassemblies. (f) Proposed self-assembly mechanism of mPEG/PS-*b*-PEI@BCNO nanoassemblies formed at different mPEG:PS feeding ratios: (i) 1:2, (ii) 1:1, and (iii) 4:1.

Furthermore, surface charge measurements confirmed successful polymer coating (Figure 5d). Bare BCNO exhibited a negative zeta potential (∼−21 mV), whereas the polymer-coated nanoassemblies displayed positive surface charges ranging from +28 to +5 mV, consistent with the presence of PEI at the particle interface. Increasing mPEG content progressively reduced the apparent surface charge, reflecting PEG-mediated shielding effects that attenuate interfacial electrostatic interactions.^55,56^

Among the formulations examined, the nanoassemblies prepared using an mPEG:PS feeding ratio of 1:2 exhibited hydrodynamic dimensions within the optimal range for nanoparticle-mediated tumor accumulation and were therefore selected for the polymer-coated assemblies exhibiting substantial mass loss between 300 and 450 °C due to polymer decomposition. Based on the residual mass fraction, the BCNO loading within the nanoassemblies was estimated to be approximately 8.3%.

### 3.7 pH-Responsive Disassembly of mPEG/PS-*b*-PEI@BCNO Nanoassemblies

The pH-responsive behavior of mPEG/PS-*b*-PEI@BCNO nanoassemblies was evaluated to verify whether the acid-labile Schiff-base linkages could mediate structural transformation under tumor-relevant acidic conditions. The nanoassemblies were incubated at pH 6.5, representing the mildly acidic tumor microenvironment, and at pH 5.0, representing the endo/lysosomal compartment, for up to 9 days. TEM analysis revealed a time-dependent disassembly of the initially spherical nanoassemblies under both acidic conditions (Figure 6a,b). The pristine nanoassemblies exhibited an average diameter of 61.03 ± 10.31 nm. Upon incubation at pH 5.0, the assemblies progressively fragmented into smaller clusters and eventually discrete nanoparticles, reaching an average diameter of 14.24 ± 4.77 nm after 9 days. A similar decrease in particle size was observed at pH 6.5, where the average diameter decreased to 13.72 ± 3.26 nm after 9 days. Although both acidic environments induced disassembly, the morphological transition occurred more rapidly at pH 5.0, consistent with accelerated hydrolysis of Schiff-base linkages under stronger acidic conditions. The disassembly process was further supported by zeta potential analysis. After 9 days of incubation at pH 6.5, the zeta potential of mPEG/PS-*b*-PEI@BCNO increased from approximately +28 mV to +36 mV (Figure S3), suggesting partial exposure of protonated PEI domains following cleavage or loosening of the polymer shell. This increase in positive surface charge is consistent with acid-induced reorganization of the nanoassemblies and may facilitate electrostatic interaction with negatively charged cellular membranes.

**Figure 6:**
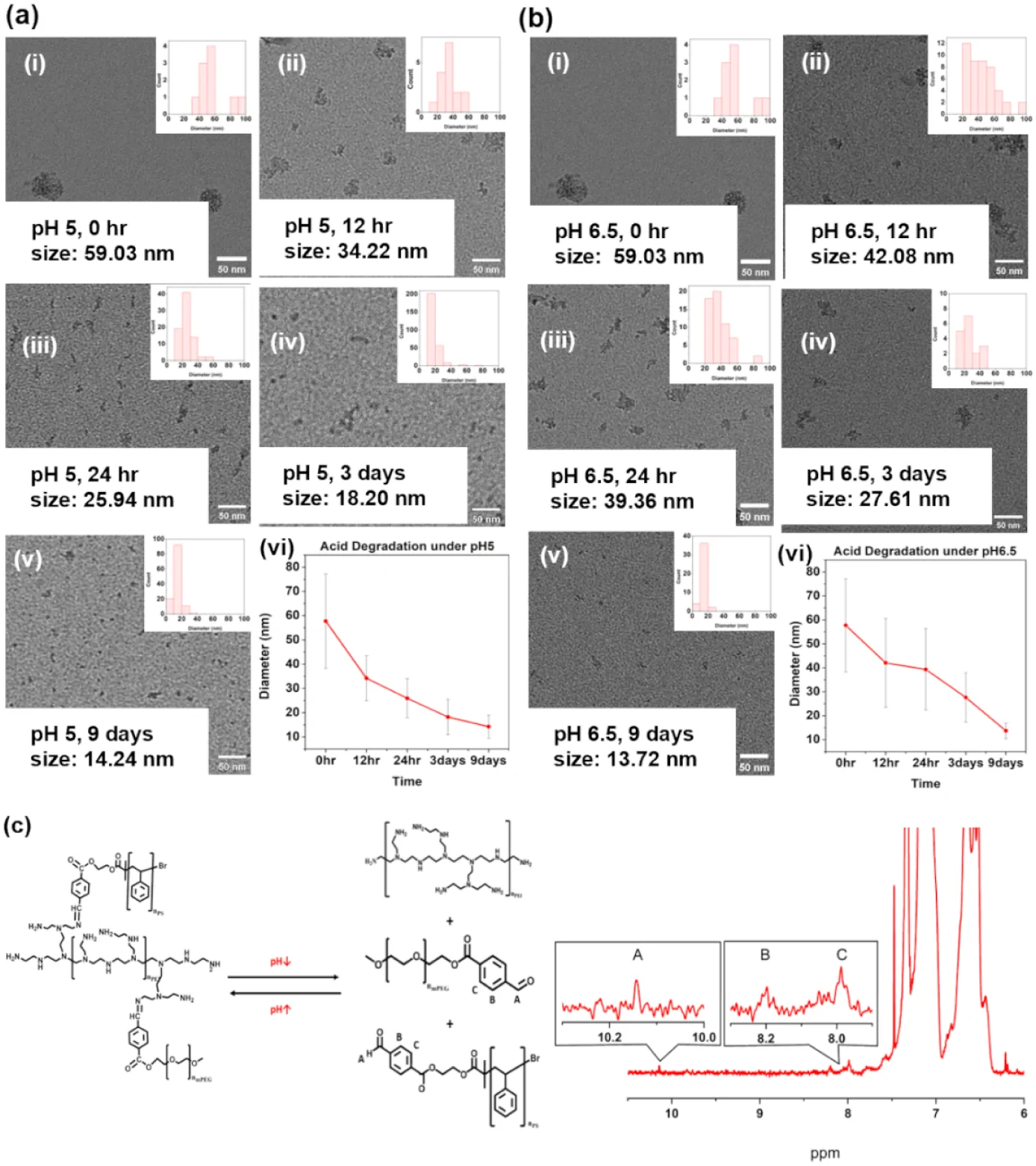
(a) TEM images of the mPEG/PS-*b*-PEI@BCNO nanoassemblies incubated in pH 5.0 buffer for various durations: (i) 0 h, (ii) 12 h, (iii) 24 h, (iv) 3 days, and (v) 9 days. (vi) Corresponding size distribution profile at pH 5.0, illustrating a progressive decrease in particle size over time under acidic conditions. (b) TEM images of the mPEG/PS-*b*-PEI@BCNO nanoassemblies incubated in pH 6.5 buffer for various durations: (i) 0 h, (ii) 12 h, (iii) 24 h, (iv) 3 days, and (v) 9 days. (vi) Corresponding size distribution profile at pH 6.5, illustrating a progressive decrease in particle size over time under acidic conditions. Scale bar: 50 nm. (c) ^1^H NMR spectrum of mPEG/PS-*b*-PEI@BCNO nanoassemblies after 9 days of incubation in pH 5.0 buffer, indicating structural change under acidic conditions. The peak observed between 7.9 and 8.2 ppm corresponds to the aromatic proton of the benzene ring in mPEG-CHO and PS-CHO.

Furthermore, ^1^H NMR spectroscopy was employed to confirm the cleavage of the Schiff base linkage between mPEG/PS and PEI segments. After incubation at pH 5.0 for 9 days, the reappearance of a characteristic aldehyde proton signal at ∼10.1 ppm was observed (Figure 6c), confirming hydrolysis of the imine bonds linking the mPEG/PS segments to PEI. In summary, the TEM, zeta-potential, and NMR results demonstrate that mPEG/PS-*b*-PEI@BCNO undergoes acid-responsive structural transformation through Schiff-base cleavage, enabling the conversion of ∼60 nm nanoassemblies into smaller BCNO-containing fragments under tumor- and lysosome-relevant acidic environments. This pH-triggered disassembly provides a key macromolecular design advantage: the nanoassemblies maintain a size regime suitable for nanoparticle delivery under neutral conditions while converting into smaller fragments under acidic conditions, which may enhance intratumoral penetration and intracellular boron release.

### 3.8 In Vitro Cytotoxicity of BCNO and mPEG/PS-*b*-PEI@BCNO Nanoassemblies

The cytotoxicity profiles of bare BCNO nanoparticles, mPEG/PS-*b*-PEI@BCNO nanoassemblies, and BPA were evaluated in UN-KC-6141 pancreatic cancer cells over a concentration range of 0–1000 μg mL^–1^ at incubation times of 24, 48, and 72 h (Figure 7a–d). All formulations exhibited time- and concentration-dependent reductions in cell viability, although their cytotoxic responses differed substantially depending on formulation architecture. Both BCNO and mPEG/PS-*b*-PEI@BCNO nanoassemblies showed concentration-dependent cytotoxicity. After 72 h, the IC_20_ values (the concentration required to inhibit 20% of cell viability) for both BCNO and mPEG/PSb-PEI@BCNO were determined to be 31.25 μg/mL, while BPA showed a substantially higher IC_20_ value of 125 μg/mL. These results indicate that incorporation of BCNO into the polymer nanoassemblies does not significantly compromise the intrinsic therapeutic activity of the boron nanomaterial.

**Figure 7:**
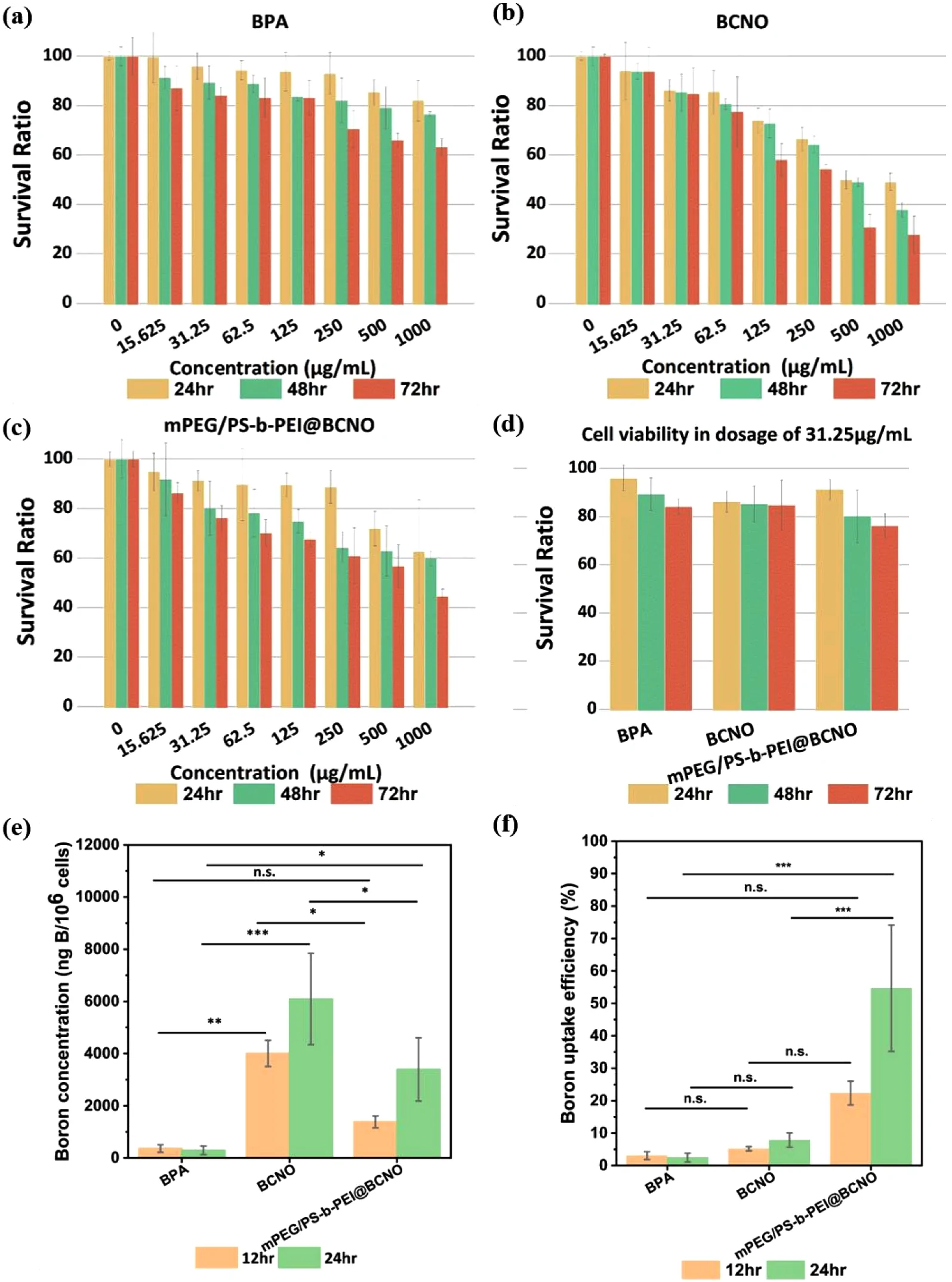
Cytotoxicity of (a) BCNO, (b) mPEG/PS-*b*-PEI@BCNO (c) BPA, and (d) at a fixed concentration of 31.25 μg/mL upon incubation with UN-KC-6141 pancreatic cancer cells for 24, 48, and 72 h. (e) ICP-MS analysis and (f) corresponding boron uptake efficiency of the intracellular boron uptake in UN-KC-6141 pancreatic cancer cells following treatment with BPA, BNCO, and mPEG/PS-*b*-PEI@BCNO nanoassemblies for 12 and 24 h. Data are presented as means ± standard deviation (SD, *n* = 3 for all experimental groups). Statistical significance was determined as follows: P* ≤ 0.05, P** ≤ 0.01, P*** ≤ 0.001.

Notably, mPEG/PS-*b*-PEI@BCNO nanoassemblies displayed slightly lower cytotoxicity compared to bare BCNO at equivalent concentrations. This attenuation is attributed primarily to the PEG-rich corona of the amphiphilic block copolymer, which reduces nonspecific nanoparticle–cell interactions and moderates direct membrane-associated toxicity. PEGylation is widely recognized for improving colloidal stability and biocompatibility while reducing undesirable cellular interactions, thereby enhancing the translational suitability of nanoparticle systems for systemic administration. Importantly, the reduced intrinsic cytotoxicity of mPEG/PS-*b*-PEI@BCNO should not be interpreted as diminished therapeutic potential. Rather, it reflects a key macromolecular design feature in which the polymer shell modulates nanoparticle biointeractions while preserving boron delivery capability.

The ability of the nanoassemblies to maintain low basal cytotoxicity while retaining effective boron delivery characteristics is advantageous for minimizing off-target effects during preirradiation exposure.

### 3.9 Cellular Boron Uptake in UN-KC-6141 Pancreatic Cancer Cells

Cellular boron uptake in UN-KC-6141 pancreatic cancer cells was quantified by ICP-MS to evaluate the delivery efficiency of mPEG/PS-*b*-PEI@BCNO nanoassemblies relative to bare BCNO nanoparticles and BPA. Based on the cytotoxicity results, the IC_20_ concentration of mPEG/PS-*b*-PEI@BCNO, 31.25 μg mL^–1^, was selected for subsequent uptake studies to ensure sufficient boron exposure while minimizing carrier-associated cytotoxicity. Cells were incubated with each boron formulation for 12 or 24 h, and the intracellular boron content was quantified (Figure 7e,f).

Both BCNO and mPEG/PS-*b*-PEI@BCNO exhibited time-dependent intracellular boron accumulation. Bare BCNO produced higher absolute boron levels, reaching 4009 and 6095 μg B per 10^6^ cells after 12 and 24 h, respectively. In comparison, mPEG/PSb-PEI@BCNO delivered 1388 and 3395 μg B per 10^6^ cells at the same time points. Although the total boron accumulation was lower for the polymer-coated nanoassemblies, this comparison must be interpreted in the context of formulation composition. TGA analysis indicated that mPEG/PS-*b*-PEI@BCNO contains approximately 8 wt % BCNO, meaning that the administered nanoassembly carries substantially less boron than an equivalent mass of bare BCNO.

When normalized to the boron content in the administered formulation, mPEG/PSb-PEI@BCNO displayed markedly higher uptake efficiency than both bare BCNO and BPA. The uptake efficiency of mPEG/PS-*b*-PEI@BCNO increased from 22.3% at 12 h to 54.66% at 24 h, whereas bare BCNO showed only 5.16% and 7.85% uptake efficiency at the corresponding time points. BPA exhibited even lower uptake efficiency, decreasing from 3.05% at 12 h to 2.45% at 24 h. These results indicate that the amphiphilic polymer coating substantially improves boron utilization efficiency, defined here as the fraction of available boron successfully delivered into cells. The enhanced delivery efficiency of mPEG/PS-*b*-PEI@BCNO is likely associated with its polymer-regulated interfacial properties. The positively charged PEI-containing shell can promote electrostatic interactions with negatively charged cellular membranes, whereas the amphiphilic mPEG/PS architecture improves colloidal dispersion and facilitates nanoparticle–cell association. Thus, although polymer coating reduces the apparent boron content per unit mass, it improves the fraction of boron that is effectively internalized.

Confocal laser scanning microscopy further supported the ICP-MS results. Because of the intrinsic green fluorescence of BCNO, intracellular localization of both BCNO and mPEG/PS-*b*-PEI@BCNO could be visualized without additional labeling. As shown in Figure 8a, green fluorescence was observed within UN-KC-6141 cells after treatment with both formulations, confirming cellular internalization. The fluorescence intensity increased with the incubation time, consistent with time-dependent uptake. Stronger fluorescence was observed in the bare BCNO group, which is in agreement with its higher absolute boron accumulation measured by ICP-MS. Importantly, all three boron formulations delivered intracellular boron levels exceeding 0.18 ng per 10^4^ cells, corresponding to approximately 10^9^ boron atoms per cell, which is a commonly cited threshold for effective BNCT. These findings demonstrate that mPEG/PS-*b*-PEI@BCNO achieves therapeutically relevant boron delivery, despite its lower intrinsic boron loading. More importantly, the results highlight a key macromolecular design advantage: polymer architecture enhances boron utilization efficiency by improving cellular delivery per available boron payload rather than relying solely on high boron content.

**Figure 8:**
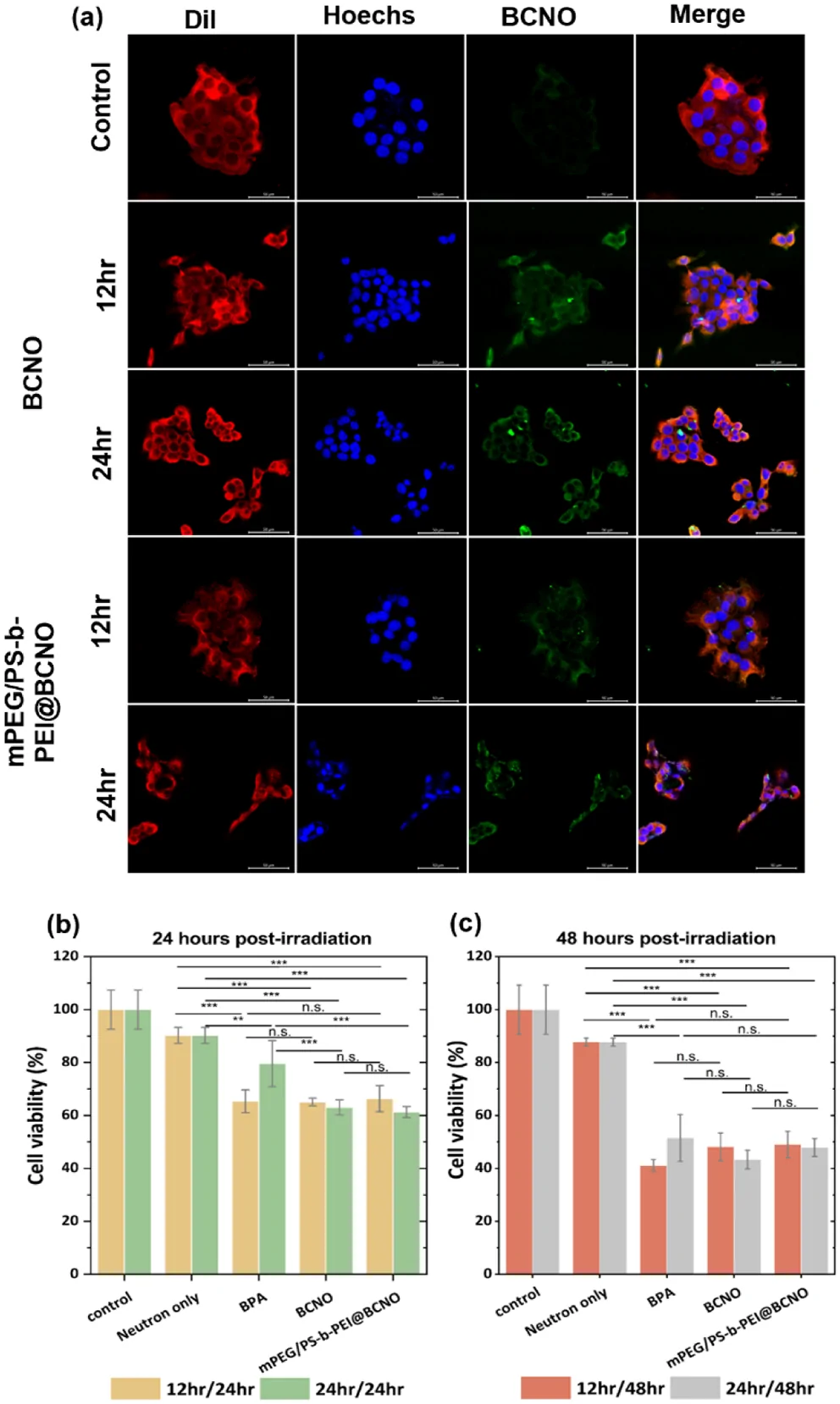
(a) Confocal laser scanning microscopy (CLSM) images of the UN-KC-6141 pancreatic cancer cells treated with BCNO and mPEG/PS-*b*-PEI@BCNO nanoassemblies at a fixed concentration of 31.25 μg/mL for 12 and 24 h. Nuclei were counterstained with Hoechst 33342 (blue), cell membranes were stained with Dil (red), and BCNO fluorescence is shown in green; scale bar: 50 μm. (b, c) Cell viability of UN-KC-6141 pancreatic cancer cells following treatment with boron agents (31.25 μg/mL) and subsequent neutron irradiation (30 min, 1.2 MW), measured at (b) 24 h and (c) 48 h post-treatment. Data are presented as means ± standard deviation (SD, *n* = 6 for all experimental groups). Statistical significance was determined as follows: P* ≤ 0.05, P** ≤ 0.01, P***≤0.001.

### 3.10 In Vitro BNCT Treatment in UN-KC-6141 Pancreatic Cancer Cells

The in vitro BNCT efficacy of mPEG/PS-*b*-PEI@BCNO was evaluated in UN-KC-6141 pancreatic cancer cells to determine whether the enhanced boron delivery efficiency observed by ICP-MS could translate into neutron-triggered cytotoxicity. Cells were incubated with BPA, bare BCNO, or mPEG/PS-*b*-PEI@BCNO at a fixed formulation concentration of 31.25 μg mL^–1^ for 12 or 24 h, followed by neutron irradiation at 1.2 MW for 30 min. Cell viability was quantified by MTS assay at 24 and 48 h after irradiation (Figure 8b,c).

The neutron-only control group showed an approximately 10% reduction in cell viability relative to the nonirradiated control, indicating a modest contribution from background radiation under the applied irradiation conditions. This effect is likely associated with low-LET secondary radiation generated during neutron irradiation, including γ-rays produced through neutron interactions with hydrogen-containing biological media. Because the magnitude of this effect was limited, the substantial decrease in viability observed in the boron-treated groups can be attributed primarily to boron-mediated neutron capture reactions.^57–59^

All boron-containing formulations produced pronounced reductions in cell viability after neutron exposure, confirming successful BNCT-mediated cytotoxicity in UN-KC-6141 cells. Cell killing increased at 48 h relative to 24 h postirradiation, consistent with progressive accumulation of radiation-induced cellular damage after neutron capture. For BPA, the 12 h incubation group showed greater cytotoxicity than the 24 h group, likely reflecting the rapid uptake and clearance kinetics of this small-molecule boron agent.^60^ In contrast, BCNO and mPEG/PS-*b*-PEI@BCNO showed slightly greater BNCT efficacy after 24 h of incubation, consistent with the time-dependent increase in intracellular boron accumulation observed by ICP-MS.

Despite differences in boron uptake profiles, no statistically significant difference in BNCT-induced cytotoxicity was observed among BPA, BCNO, and mPEG/PS-b-PEI@BCNO under these 2D monolayer conditions. This result should be interpreted in the context of the experimental model. In 2D cultures, all cells are directly exposed to formulations and experience minimal diffusion barriers; therefore, differences in nanoparticle penetration, pH-responsive disassembly, and microenvironment-dependent transport are not fully represented. Under such simplified conditions, intracellular boron levels above the therapeutic threshold may be sufficient to produce comparable BNCT responses across the formulations.

Importantly, the comparable performance of mPEG/PS-*b*-PEI@BCNO should not be viewed as a limitation of the nanoassembly design. Rather, it indicates that the polymer-coated formulation retains BNCT activity despite its substantially lower intrinsic BCNO content. This finding supports the central design concept that macromolecular architecture can improve boron utilization efficiency per available boron payload. The advantages of mPEG/PS-*b*-PEI@BCNO—namely colloidal stabilization, polymer-regulated cellular interaction, and acid-responsive disassembly—are expected to become more relevant in transport-limited tumor models, where extracellular matrix density, pH gradients, and heterogeneous nanoparticle penetration critically influence therapeutic outcome.

Although mPEG/PS-*b*-PEI@BCNO did not demonstrate superior cytotoxicity compared to BPA or bare BCNO in 2D monolayer cultures, its design features remain highly relevant for in vivo applications. The lack of enhanced efficacy in 2D models does not preclude its potential benefit, as such models fail to capture critical aspects of the tumor microenvironment—including extracellular matrix density, pH gradients, and limited nanoparticle transport. These features can dramatically influence nanoparticle behavior, particularly in terms of stability, cellular uptake, and boron release. The amphiphilic polymer shell of mPEG/PS-*b*-PEI@BCNO offers enhanced colloidal stability, prolonged circulation, and pH-responsive disassembly, which may provide significant therapeutic advantages under more physiologically relevant conditions. These findings justify further evaluation in 3D spheroids, where the contribution of macromolecular transport and pH-responsive nanoassembly behavior can be more rigorously assessed.

### 3.11 Cellular Uptake and Penetration of BCNO-Based Nanoassemblies in 3D Tumor Spheroids

To evaluate whether the polymer architecture facilitates nanoparticle transport in a physiologically relevant tumor model, the penetration behavior of bare BCNO and mPEG/PS-*b*-PEI@BCNO nanoassemblies was examined in UN-KC-6141 pancreatic tumor spheroids. Spheroids with an average diameter of approximately 400 μm after 4 days of culture (Figure S4) were incubated with BCNO or mPEG/PS-*b*-PEI@BCNO at 31.25 μg mL^–1^ for 6, 12, 24, and 72 h under physiological pH (7.4) or mildly acidic pH (6.5). The intrinsic green luminescence of BCNO enabled direct visualization by confocal laser scanning microscopy (CLSM), while nuclei were counterstained with Hoechst 33342. Optical sections were acquired at 5 μm intervals to assess nanoparticle distribution across spheroid cross sections.

As shown in Figure 9, CLSM imaging of 3D UN-KC-6141 pancreatic tumor spheroids revealed distinct pH- and formulation-dependent distribution profiles for bare BCNO and mPEG/PS-*b*-PEI@BCNO. At pH 7.4, bare BCNO nanoparticles showed broader fluorescence across the spheroid sections than mPEG/PS-*b*-PEI@BCNO, indicating greater inward penetration (Figure 9a,b). This is likely due to the smaller primary size of bare BCNO, which facilitates diffusion through the compact spheroid matrix.^61^ The line profile analysis supports this observation, showing a wider fluorescence distribution for bare BCNO compared to the more localized signal of mPEG/PS-*b*-PEI@BCNO. In contrast, mPEG/PS-*b*-PEI@BCNO at pH 7.4 was mainly retained near the spheroid surface or the basal region, appearing as discrete fluorescent clusters. This restricted penetration is consistent with its larger assembled size and possible PEI-mediated association with negatively charged cellular or extracellular components at the spheroid periphery, which may enhance surface retention but limit inward diffusion.^62,63^

**Figure 9:**
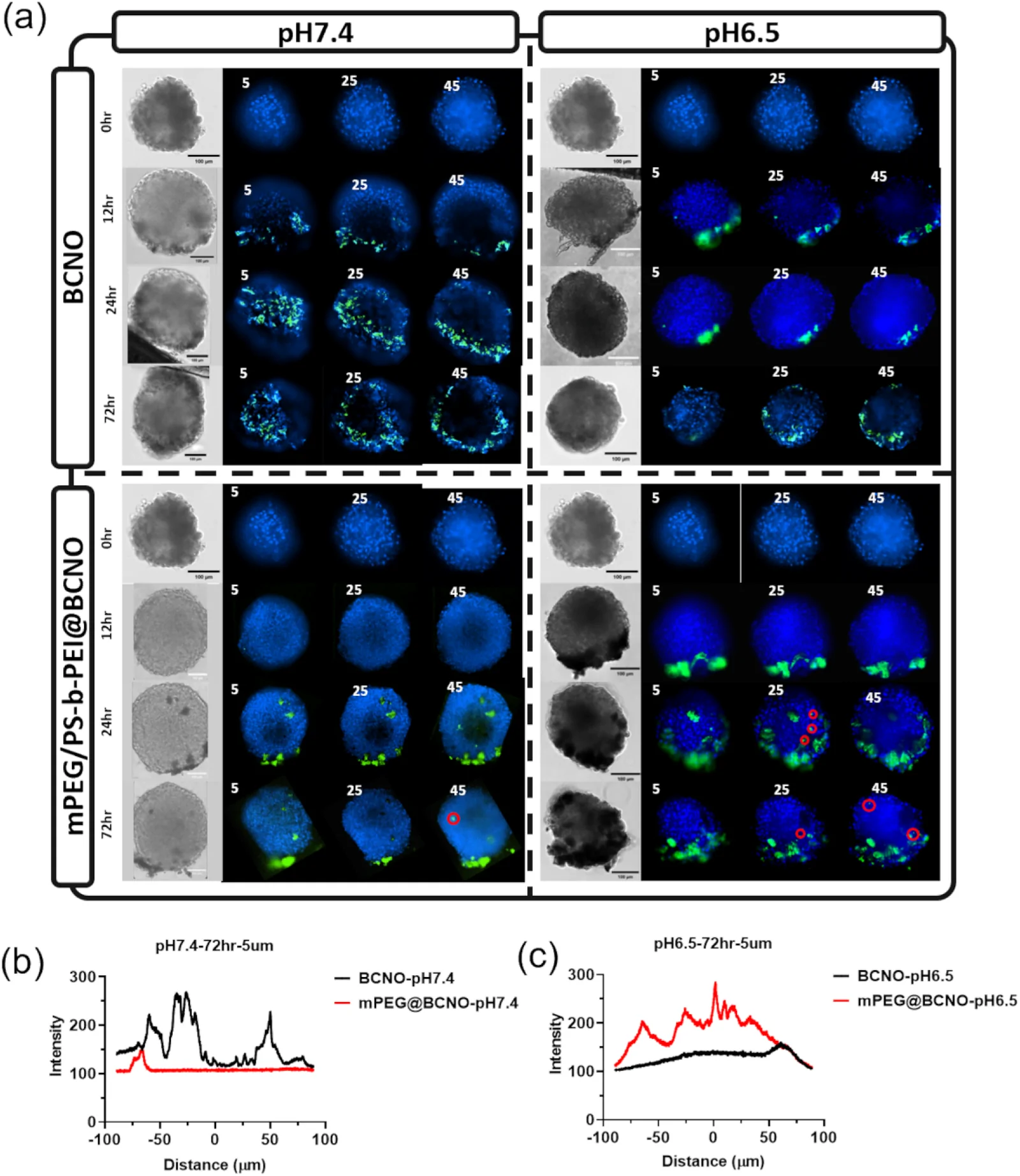
(a) Confocal laser scanning microscopy (CLSM) imaging of the 3D spheroids of UN-KC-6141 cells treated with BCNO and mPEG/PS-*b*-PEI@BCNO at a fixed concentration of 31.25 μg/mL for 6, 12, 24, and 72 h in pH 7.4 and pH 6.5. Nuclei were counterstained with Hoechst 33342 (blue), and BCNO fluorescence is shown in green; scale bar: 100 μm. Stacked CLSM line profile analysis of bare BCNO nanoparticles and mPEG/PS-*b*-PEI@BCNO in 3D tumor spheroids at (b) pH 7.4 and (c) pH 6.5.

At pH 6.5, bare BCNO exhibited reduced penetration relative to pH 7.4, with fluorescence becoming more localized at the spheroid surface. This may result from pH-dependent changes in surface charge or colloidal stability, leading to partial aggregation and reduced diffusion through the spheroid. For mPEG/PS-*b*-PEI@BCNO at pH 6.5, the CLSM line profile showed a broad and irregular fluorescence distribution with multiple localized intensity maxima across the spheroid section (Figure 9). This pattern differs from a purely peripheral signal and agrees with the CLSM images showing discrete fluorescent clusters within the spheroid core at 5, 25, and 45 μm depths (Figure 9a). These observations suggest that acidic pH promoted partial inward redistribution of BCNO-containing species, consistent with Schiff-base cleavage and disassembly of the nanoassemblies into smaller fragments. These smaller species are expected to diffuse more efficiently through the dense multicellular matrix.

Because the spheroids were cultured in static ultralow-attachment U-bottom plates, gravitational sedimentation may contribute to enhanced nanoparticle exposure at the basal spheroid region. This effect was most apparent for bare BCNO, where the fluorescence was enriched near the lower portion of the spheroids. To reduce positional bias, nanoparticle suspensions were gently resuspended before incubation, and the fluorescence distribution was evaluated across multiple optical sections. Nevertheless, this limitation should be acknowledged when interpreting the absolute penetration depth from CLSM images. In summary, 3D spheroid results show that bare BCNO is largely restricted to peripheral regions, whereas mPEG/PS-*b*-PEI@BCNO exhibits pH-dependent redistribution and partial inward penetration. These findings support the central design rationale that acid-labile polymer nanoassemblies can couple extracellular stability with microenvironment-triggered size transformation, thereby improving boron transport under tumor-relevant conditions.

### 3.12 Quantitative Boron Uptake in 3D Tumor Spheroids

In addition to confocal laser scanning microscopy (CLSM), inductively coupled plasma mass spectrometry (ICP-MS) was employed to quantitatively assess the boron uptake in 3D tumor spheroids. Spheroids with an average diameter of approximately 400 μm, formed after 4 days of culture, were treated for 72 h under two dosing conditions: (1) a fixed formulation concentration and (2) a normalized ^10^B concentration. In the fixed-concentration experiment, spheroids were incubated with BPA, bare BCNO, or mPEG/PS-*b*-PEI@BCNO at 31.25 μg mL^–1^. In the normalized-boron experiment, the dosing concentration was adjusted to provide an equivalent ^10^B concentration of approximately 0.1863 μg ^10^B mL^–1^, corresponding to 31.25 μg mL^–1^ mPEG/PS-*b*-PEI@BCNO, 2.5 μg mL^–1^ BCNO, and 3.26 μg mL^–1^ BPA. For BCNO and mPEG/PSb-PEI@BCNO, uptake was evaluated under both physiological pH 7.4 and mildly acidic pH 6.5 to examine the influence of tumor-relevant acidity on boron delivery.

Under a fixed formulation concentration, both nanoparticle-based systems delivered substantially higher boron levels than BPA (Figure 10a,b). BPA showed minimal spheroid uptake, approximately 25 μg ^10^B/g tissue, whereas bare BCNO produced the highest absolute boron accumulation, reaching approximately 322 and 300 μg ^10^B g^–1^ tissue at pH 7.4 and 6.5, respectively. This result is expected because bare BCNO contains a much higher intrinsic boron fraction than polymer-coated nanoassemblies. In comparison, mPEG/PS-*b*-PEI@BCNO achieved boron levels of approximately 185 μg ^10^B g^–1^ tissue at pH 7.4 and 120 μg ^10^B g^–1^ tissue at pH 6.5. Although the absolute boron accumulation was lower than that of bare BCNO, this comparison must be interpreted in light of the nanoassembly composition: TGA analysis showed that mPEG/PS-*b*-PEI@BCNO contains only approximately 8 wt % BCNO.

**Figure 10:**
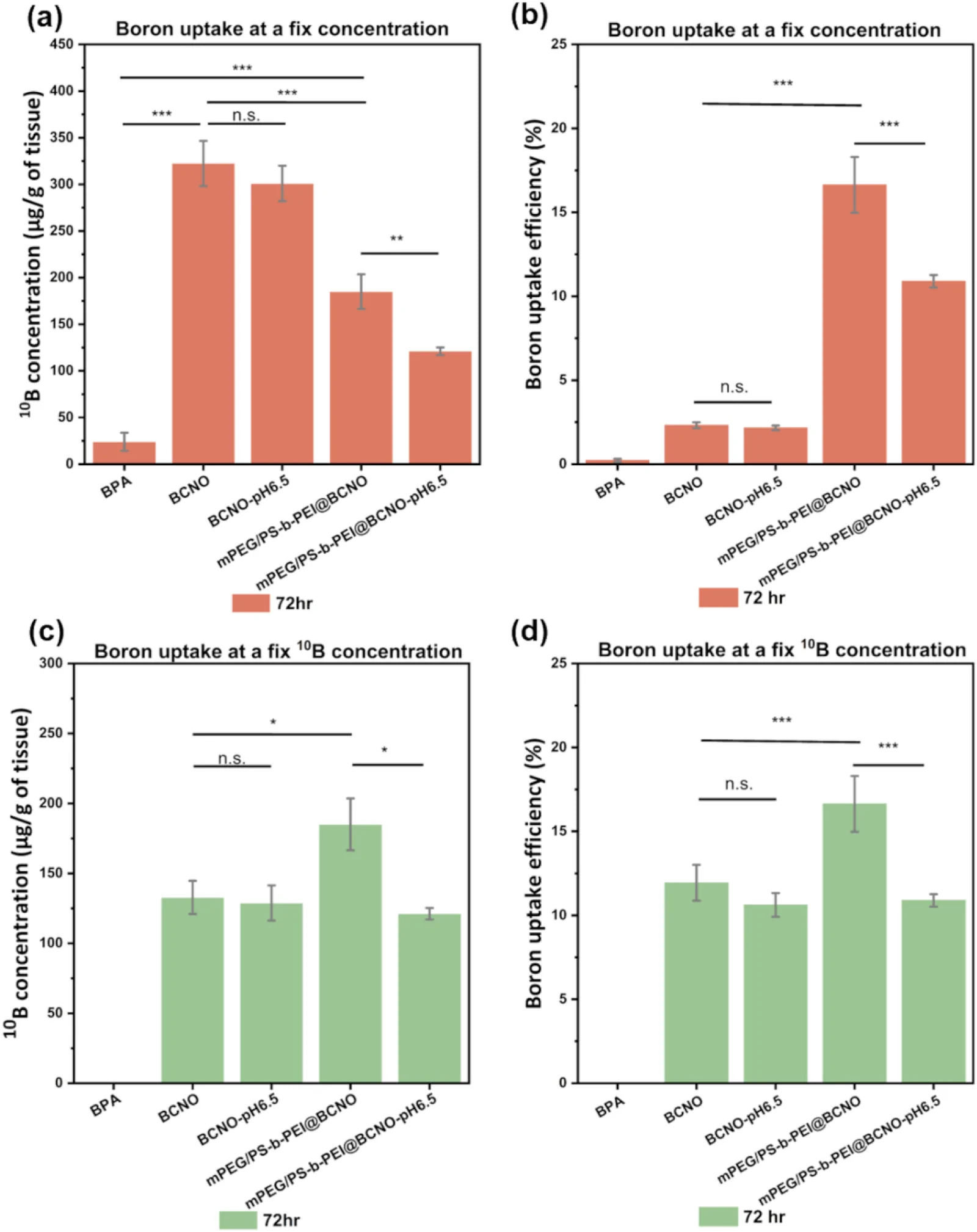
(a) Intracellular boron accumulation measured by ICP-MS and (b) corresponding boron uptake efficiency in UN-KC-6141 pancreatic cancer cell spheroids after 72 h treatment with BPA, BNCO, and mPEG/PS-*b*-PEI@BCNO nanoassemblies at a fixed concentration of 31.25 μg/mL. (c) ICP-MS analysis and (d) boron uptake efficiency under conditions with equalized ^10^B concentrations for each treatment group. Data are presented as means ± standard deviation (SD, *n* = 3 for all experimental groups). Statistical significance was determined as follows: P* ≤ 0.05, P** ≤ 0.01, P*** ≤ 0.001.

To clarify this distinction, the boron uptake efficiency was calculated by normalizing spheroid boron accumulation to the amount of boron available in each formulation. Under fixed-concentration dosing, mPEG/PS-*b*-PEI@BCNO exhibited markedly higher uptake efficiency, reaching 16.64% at pH 7.4 and 10.89% at pH 6.5, compared to 2.32% and 2.17% for bare BCNO under the same pH conditions. BPA showed only 0.23% uptake efficiency. These results demonstrate that the amphiphilic mPEG/PS-*b*-PEI coating substantially improves the boron utilization efficiency in 3D spheroids, even though it dilutes the apparent boron content of the administered material.

The enhanced uptake efficiency of mPEG/PS-*b*-PEI@BCNO is attributed to its polymer-regulated interfacial properties. The positively charged PEI-containing domains likely promote electrostatic interactions with negatively charged cellular membranes, thereby enhancing spheroid association and cellular internalization.^64,65^ In addition, the amphiphilic polymer architecture improves colloidal organization and may facilitate prolonged contact with the spheroid surface.^66–68^ In contrast, BPA, as a small-molecule boron agent, exhibited limited retention in the 3D spheroid matrix under the experimental conditions.

The response under mildly acidic conditions was more complex. At pH 6.5, mPEG/PS-*b*-PEI@BCNO underwent acid-triggered fragmentation into smaller BCNO-containing species; however, the total boron accumulation and BNCT efficacy were lower than those observed at pH 7.4. This finding indicates that size reduction alone does not necessarily improve the productive boron delivery. From a particokinetic perspective, nanoparticle accumulation in 3D spheroids depends on coupled processes, including diffusion, sedimentation-dependent exposure, cell-surface association, internalization, and intracellular retention, rather than diffusional mobility alone.^65–67^ Although smaller fragments may diffuse more readily within the spheroid matrix, they may also have reduced sedimentation-driven contact, weaker multivalent membrane association, and lower endocytic retention compared with the more intact nanoassemblies at pH 7.4^65,67^. These effects are further influenced by the diffusion barriers and spatial gradients present in 3D tumor spheroids.^68,69^ Therefore, the reduced BNCT efficacy at pH 6.5 is consistent with the lower total boron retention measured by ICP-MS. These results indicate that acid-responsive disassembly modulates spheroid transport and spatial redistribution but does not necessarily increase the total boron accumulation under all conditions.

To directly compare delivery efficiency independent of formulation boron content, spheroids were treated with equivalent ^10^B concentrations (Figure 10c,d). Under these conditions, mPEG/PS-*b*-PEI@BCNO produced higher boron accumulation than bare BCNO at pH 7.4, confirming that polymer coating improves spheroid internalization efficiency when boron availability is normalized. At pH 6.5, the uptake efficiencies of mPEG/PS-*b*-PEI@BCNO and BCNO became more comparable at 10.89% and 10.62%, respectively, suggesting that acid-induced disassembly partially reduces the uptake advantage conferred by the intact cationic nanoassembly. In contrast, BPA uptake was below the ICP-MS detection limit under the normalized-boron condition, further highlighting the limited retention of small-molecule boron agents in this 3D model. Importantly, mPEG/PS-*b*-PEI@BCNO achieved boron concentrations well above the commonly accepted minimum therapeutic threshold for BNCT, approximately 20 μg ^10^B g^–1^ tissue, under both pH conditions. This result demonstrates that the nanoassemblies can deliver therapeutically relevant boron levels to dense multicellular tumor spheroids despite their relatively low BCNO content. More broadly, the data support a central macromolecular design principle: boron delivery performance should not be evaluated solely by intrinsic boron loading, but also by boron utilization efficiency, which is governed by particle architecture, surface charge, pH responsiveness, and transport behavior in tumor-like tissue models.

The present study is intentionally framed as a macromolecular design and 3D tumor-model validation study rather than an in vivo therapeutic evaluation. The findings establish how amphiphilicity, electrostatic complexation, and acid-labile Schiff-base chemistry collectively govern BCNO nanoassembly formation, pH-responsive disassembly, spheroid penetration, and boron utilization efficiency. This manuscript highlights the structure–function relationships at the polymer–nanoparticle interface and demonstrates their relevance in physiologically motivated 3D tumor spheroids. While in vivo biodistribution and BNCT efficacy remain important for translational next steps, they require separate optimization of pharmacokinetics, administration route, radiation geometry, and dosimetry. These studies will be addressed in a subsequent manuscript and are outside the scope of the present work.

### 3.13 In Vitro 3D Spheroid BNCT Treatment

The therapeutic efficacy of mPEG/PS-*b*-PEI@BCNO was further evaluated in 3D UN-KC-6141 tumor spheroids to determine whether the enhanced boron utilization observed in the spheroid uptake studies could translate into improved BNCT response. Spheroids with an average diameter of approximately 400 μm after 4 days of culture were incubated with boron formulations for 72 h before neutron irradiation. Two dosing strategies were used: a fixed concentration and a normalized ^10^B concentration. In the fixed-concentration experiment, spheroids were treated with BPA, BCNO, or mPEG/PS-*b*-PEI@BCNO at 31.25 μg/mL. In the normalized-^10^B experiment, the administered doses were adjusted to provide an equivalent ^10^B concentration of approximately 0.1863 μg/mL, corresponding to 31.25 μg/mL of mPEG/PS-*b*-PEI@BCNO, 2.5 μg/mL of BCNO, and 3.26 μg/mL of BPA. BCNO and mPEG/PS-*b*-PEI@BCNO were evaluated under both physiological (pH 7.4) and mildly acidic (pH 6.5) conditions to assess the influence of tumor-relevant acidity on BNCT efficacy. After incubation, spheroids were exposed to neutron irradiation at 1.2 MW for 30 min, and the cell viability was quantified by MTS assay 24 h postirradiation.

Under physiological pH, the neutron-only control showed only a modest decrease in cell viability of approximately 5% relative to the nonirradiated control (Figure 11), indicating that neutron exposure alone produced limited cytotoxicity under the applied irradiation conditions.^57–59^ In contrast, all boron-treated groups exhibited substantial BNCT-mediated spheroid growth inhibition, confirming that the observed therapeutic response arose primarily from boron-dependent neutron capture reactions. At a fixed boron agent concentration of 31.25 μg/mL, all three treatment groups—BPA, BCNO, and mPEG/PS-*b*-PEI@BCNO—induced cell death levels of approximately 35%, 57%, and 52%, respectively. Although the differences among the three boron formulations were not statistically significant under this dosing condition, the result is notable because mPEG/PS-*b*-PEI@BCNO achieved a therapeutic effect comparable to bare BCNO despite containing only ∼8 wt % BCNO. This finding indicates that the polymer nanoassembly substantially improves the functional use of its available boron payload and that its lower intrinsic boron loading does not preclude effective BNCT activity in a transport-limited 3D tumor model.

**Figure 11:**
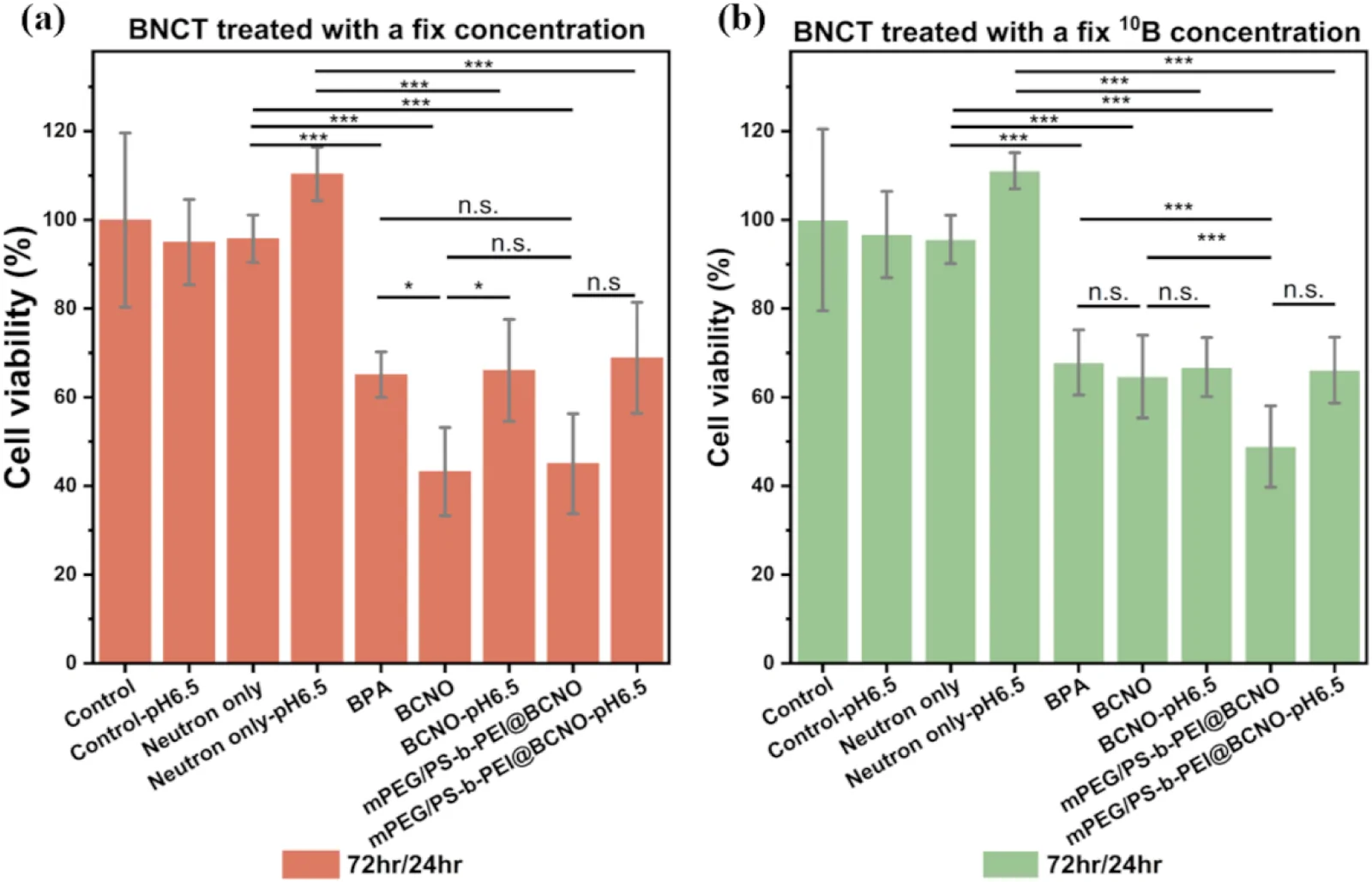
Cell viability of UN-KC-6141 pancreatic tumor spheroids following treatment with BPA, BNCO, and mPEG/PS-*b*-PEI@BCNO nanoassemblies under two conditions: (a) fixed boron agent concentration of 31.25 μg/mL and (b) equivalent ^10^B concentration, followed by neutron irradiation (30 min at 1.2 MW). Data are presented as means ± standard deviation (SD, *n* = 6 for all experimental groups). Statistical significance was determined as follows: P* ≤ 0.05, P** ≤ 0.01, P*** ≤ 0.001.

A clearer distinction emerged when the formulations were compared at equivalent ^10^B concentration. Under normalized boron dosing, mPEG/PS-*b*-PEI@BCNO produced the highest BNCT efficacy, inducing approximately 52% cell death, compared with 36% for BCNO and 33% for BPA (*P* ≤ 0.001). This statistically significant enhancement indicates that mPEG/PS-*b*-PEI@BCNO facilitates more efficient boron delivery and cellular internalization under physiological conditions. Despite requiring a higher agent concentration to match the fixed ^10^B levels due to its relatively low boron loading, mPEG/PS-*b*-PEI@BCNO demonstrated significantly enhanced therapeutic efficacy (*P* ≤ 0.001) compared to both BCNO and BPA. This statistically significant enhancement demonstrates that the nanoassembly delivers boron more effectively on a per-boron basis than either bare BCNO or BPA. The improved therapeutic response is consistent with the ICP-MS results, which showed higher boron uptake efficiency for mPEG/PS-*b*-PEI@BCNO in 3D spheroids. These data support the central design premise that polymer architecture can enhance boron utilization efficiency by improving cellular association, spheroid retention, and intracellular delivery rather than relying solely on high intrinsic boron content. This design implication is significant: by increasing the fraction of boron that is productively delivered to tumor cells, effective BNCT may be achieved with a lower administered drug dose, thereby reducing off-target boron exposure, systemic toxicity, and material burden. Thus, boron utilization efficiency, rather than boron loading alone, should be considered a key criterion in the design of next-generation polymer-based BNCT agents.

The response under mildly acidic conditions was more complex. At pH 6.5, the overall BNCT efficacy of the boron-treated groups was reduced relative to that at pH 7.4, despite the pH-responsive disassembly of mPEG/PS-*b*-PEI@BCNO. This attenuation is consistent with the lower total boron accumulation observed by ICP-MS under the acidic conditions. As discussed above, acid-triggered disassembly may generate smaller, more diffusive fragments that can penetrate deeper into the spheroid but exhibit reduced sedimentation, shorter membrane residence time, and less efficient membrane wrapping. In parallel, a mildly acidic extracellular pH may alter membrane fluidity, protein conformation, and endocytic activity, thereby reducing nanoparticle internalization. These coupled effects likely reduce the total boron payload retained within the spheroids and diminish the resulting BNCT response.

The unexpectedly higher viability observed in the pH 6.5 neutron-only group further suggests that acidic stress may alter the cellular response to irradiation.^70^ Mild extracellular acidosis can activate adaptive survival pathways, including MAPK signaling and autophagy, which are known to support stress tolerance and treatment resistance in tumor cells.^71,72^ Therefore, the acidic spheroid environment may not simply promote nanoassembly disassembly; it may also induce cellular phenotypes that are less susceptible to radiation-induced damage.^73^ This observation highlights the importance of evaluating BNCT agents in 3D models that recapitulate microenvironmental stress rather than relying exclusively on 2D monolayer cultures.

In summary, the 3D spheroid BNCT results demonstrate that mPEG/PS-*b*-PEI@BCNO is an efficient boron delivery platform capable of producing significant neutron-triggered cytotoxicity in a physiologically relevant multicellular tumor model. Its superior performance under equivalent ^10^B dosing establishes a key structure–function relationship: the amphiphilic, cationic, and pH-responsive polymer architecture improves boron utilization efficiency and therapeutic output per available boron payload. Although acidic conditions reduced overall efficacy, this result provides mechanistic insight into how tumor microenvironmental acidity modulates both nanoassembly behavior and cellular treatment response. These findings define a clear macromolecular design framework for further optimization of boron loading, disassembly kinetics, and tumor penetration.

## 4 Conclusion

In this study, we established mPEG/PS-*b*-PEI@BCNO as a structurally adaptive polymer–inorganic nanoassembly platform for boron delivery in BNCT. Beyond developing a boron nanocarrier, this work provides a systematic investigation of polymer-directed BCNO nanoparticle self-assembly, an area that remains underexplored for boron-based inorganic nanomaterials. The amphiphilic mPEG/PS-*b*-PEI copolymer was constructed through controlled radical polymerization, aldehyde end-functionalization, and acid-labile Schiff-base coupling with PEI. This modular architecture integrates three complementary polymer domains: mPEG for colloidal stabilization and interfacial shielding, PS for hydrophobic assembly integrity, and PEI for electrostatic complexation with negatively charged BCNO nanoparticles and cellular membranes. The self-assembly study revealed that the hydrophobic–hydrophilic balance of the copolymer critically governs BCNO nanoparticle organization. By varying the mPEG/PS ratio, the morphology, size, packing density, and surface charge of the resulting nanoassemblies could be systematically regulated. Higher PS content promoted compact spherical nanoassemblies, whereas increased mPEG fraction reduced hydrophobic association and favored smaller clusters or more discrete nanoparticle structures. The optimized formulation formed uniform spherical nanoassemblies of approximately 61 nm with positive surface charge and ∼8 wt % BCNO content. Under acidic conditions relevant to the tumor microenvironment and endo/lysosomal compartments, Schiff-base cleavage induced structural transformation into smaller BCNO-containing fragments, demonstrating that the polymer architecture confers both assembly control and stimuli-responsive adaptability. In vitro studies demonstrated that mPEG/PS-*b*-PEI@BCNO enhances boron utilization efficiency rather than relying solely on high boron loading. In 2D UN-KC6141 pancreatic cancer cells, the nanoassemblies showed reduced basal cytotoxicity and higher boron uptake efficiency than bare BCNO and BPA while retaining effective BNCT activity. In 3D tumor spheroids, mPEG/PS-*b*-PEI@BCNO delivered boron above the therapeutic threshold and, under equivalent ^10^B dosing, produced the strongest BNCT response, inducing approximately 52% cell death and significantly outperforming both BCNO and BPA. Overall, this work defines a macromolecular design framework in which amphiphilic polymer architecture regulates BCNO nanoparticle assembly, pH-responsive transformation, and boron delivery function. The findings identify boron utilization efficiency as a key design criterion for next-generation polymer-based BNCT nanodrugs.

## Supplementary Material



## Notes

Photoluminescence and zeta potential characterization of BCNO nanoparticles; XPS elemental composition of BCNO; ^1^H NMR analysis of mPEG-CHO and copolymer composition; zeta potential changes of mPEG/PS-*b*-PEI@BCNO and BCNO after incubation at pH 6.5; and representative UN-KC-6141 spheroid images and quantitative analysis of spheroid size (PDF)
