# Supplementary material for: Polymer-Directed Self-Assembly of BCNO Nanoparticles for Efficient Boron Utilization in Boron Neutron Capture Therapy
Source: ACS Nanosci Au. 2026 Sep 26;6(5):954–75. doi: 10.1021/acsnanoscienceau.6c00088 (PMC13693259; doi:10.1021/acsnanoscienceau.6c00088)
Supplement: Supplementary file 1 [file ng6c00088_si_001.pdf]

Polymer-Directed Self-Assembly of BCNO Nanoparticles for Efficient Boron Utilization in Boron Neutron Capture Therapy

Wei-Hsin Chan[1], Lu Ang Chen[1], Wei-Yuan Huang[1], Lin-Sheng Yu[2], Tzu-Chieh Sun[3], Fang-Hsin Chen[3,4], Chi-Shuo Chen[2,3], Tzu-Wei Wang[1,5], **Pei-Yuin Keng[1,3*]**

[1]Department of Material Science and Engineering, National Tsing Hua University, Hsinchu City 300, Taiwan

[2]Department of Biomedical Engineering and Environmental Sciences, National Tsing Hua University, Hsinchu City 300, Taiwan

[3]Boron Neutron Capture Therapy (BNCT) center, National Tsing Hua University, Hsinchu City 300, Taiwan

[4]Insititute of Nuclear Engineering and Science, National Tsing Hua University, Hsinchu

City 300, Taiwan

[5]Institute of Oral Biology, National Yang Ming Chiao Tung University, Taipei 112304, Taiwan

Correspondence: keng.py@mx.nthu.edu.tw

Supporting information:

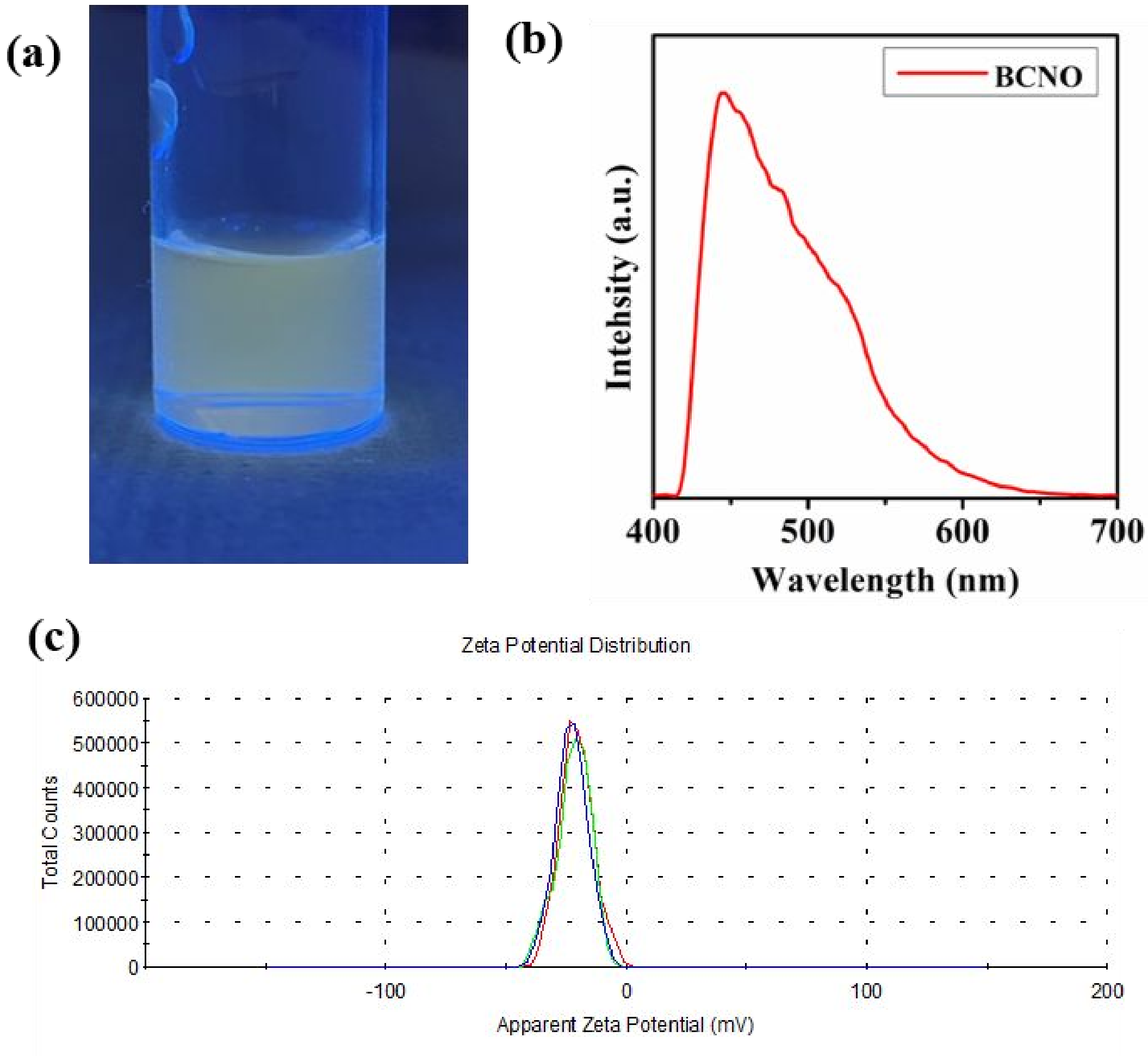

Figure S1. (a) Photoluminescence of BCNO nanoparticles, showing a broad emission peak centered at approximately 450 nm under excitation at 365 nm. (b) Photoluminescence of BCNO nanoparticles dispersed in aqueous solution under UV irradiation at an excitation wavelength of 365 nm. (c) Surface zeta potential measure of BCNO nanoparticles.

Table S1. Elemental composition of BCNO nanoparticles as determined by X-ray photoelectron spectroscopy (XPS).

	**B%**	**C%**	**N%**	**O%**
BCNO	37.26	7.29	27.18	28.27

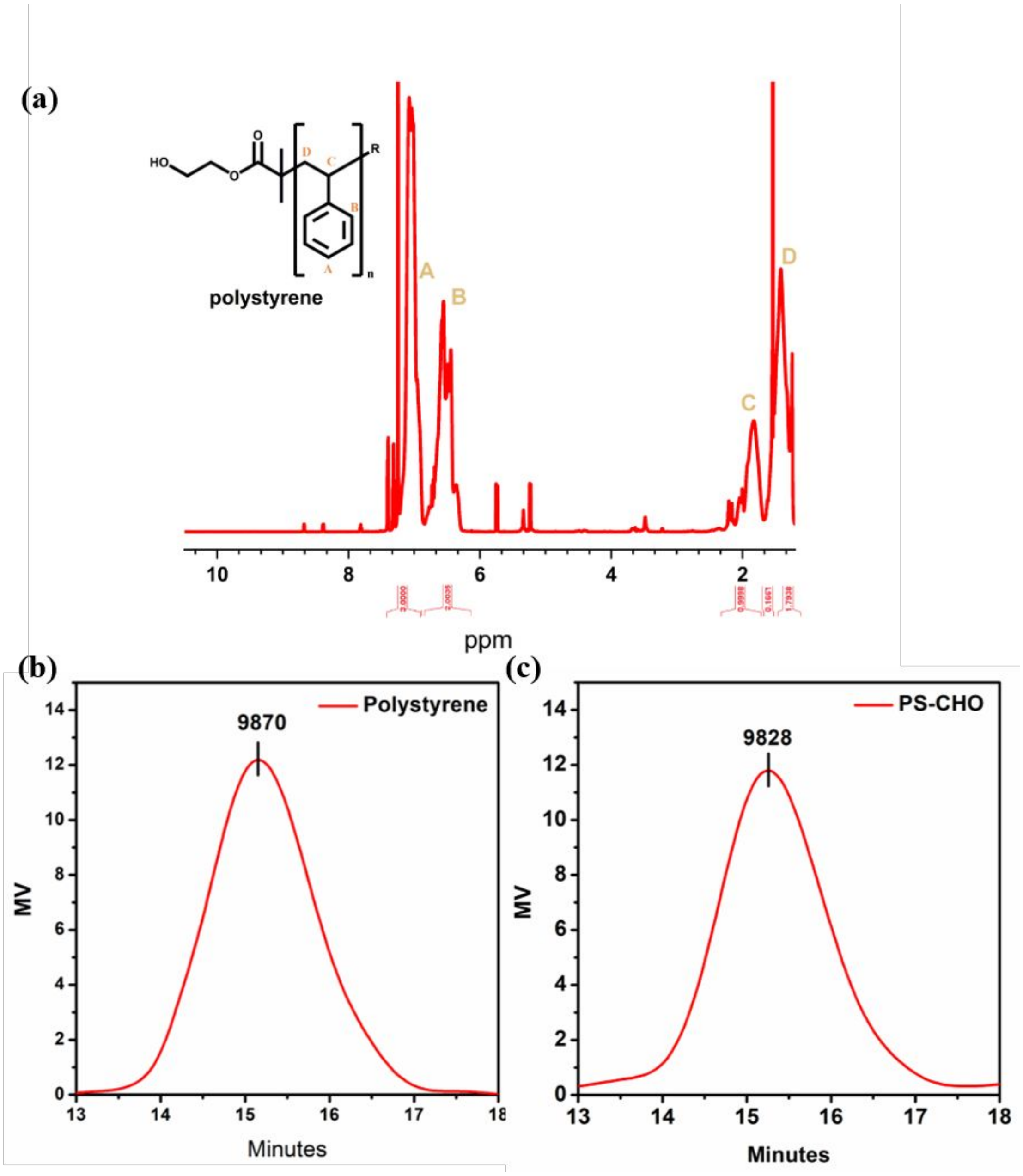

Figure S2. (a) ^{1}H NMR spectrum of polystyrene and (b-c) Gel permeation chromatography (GPC) trace of (b) polystyrene (PS) and(c) benzaldehyde end-functionalized polystyrene (PS-CHO).

End-group functionalization efficiency determined by ^{1}H NMR integration

End-group functionalization efficiencies were estimated from normalized ^{1}H NMR integration of diagnostic aldehyde signals relative to polymer backbone resonances. For

mPEG-CHO, the aldehyde proton at ~10.1 ppm was used as the end-group signal, while the PEG backbone resonance at ~3.5–3.8 ppm was used as the polymer-chain reference. The aromatic protons of the 4-formylbenzoate end group at ~7.9–8.2 ppm provided supporting structural confirmation and showed the expected internal end-group integration relationship with the aldehyde proton.

The functionalization efficiency of mPEG-CHO was calculated according to the following:

Functionalization efficiency of mPEG-CHO (%) = $\frac{I_{CHO}/1}{I_{PEG}/4n_{EO}} x\ 100\%$

Where n_{eo} = 114;, I_{CHO} is the integration of the aldehyde proton, I_{PEG} is the integral of PEG backbone resonance, and n_{EO} is the number of ethylene oxide repeat units in mPEG. For mPEG 5K, n_{EO} was approximated as 114, corresponding to $4n_{neo}$ = 456 PEG backbone protons per polymer chain.

Functionalization efficiency of mPEG-CHO (%) = $\frac{456 I_{CHO}/1}{I_{PEG}/4n_{EO}} x\ 100\%$.

Determination of Copolymer Composition by ¹H NMR Analysis

The molar composition of the mPEG/PS-b-PEI copolymer was determined by quantitative integration of ¹H NMR signals assigned to the ethylene protons of mPEG, the aromatic protons of polystyrene, and the methylene protons of hyperbranched PEI. The relative molar ratio of each segment was calculated according to the following relationship:

$$\text{mPEG:PS:PEI} = \frac{\mathrm{I_b}}{4 \times 113} : \frac{I_c + I_d}{5 \times n_{PS}} : \frac{I_a}{4 \times 41.47}$$

where I_b corresponds to the integrated ethylene proton signal of mPEG, I_c and I_d represent the aromatic proton signals of the PS segments, and I_a denotes the methylene proton signal of PEI. The parameter *n*PS represents the degree of polymerization of the polystyrene block, as determined by NMR analysis prior to conjugation. Using this method, the copolymer composition was estimated to be approximately mPEG:PS:PEI = 1:1:2.

For copolymers prepared using alternative feeding ratios, the molar fractions of polystyrene (*f*PS), mPEG (*f*mPEG), and PEI (*f*PEI) were calculated based on the molar contributions of each segment:

$$f_{PS} = \frac{M_{PS}}{M_{mPEG}+M_{PS}+M_{PEI}} \quad ; \quad f_{mPEG} = \frac{M_{mPEG}}{M_{mPEG}+M_{PS}+M_{PEI}} \quad ; \quad f_{PEI} = \frac{M_{PEI}}{M_{mPEG}+M_{PS}+M_{PEI}}$$

where M_{PS}, M_{mPEG}, and M_{PEI} represent the molar amounts of each segment derived from NMR integration. The calculated compositions for all copolymers are summarized in Figure 4c and were used to analyze the influence of copolymer architecture on nanoassembly behavior.

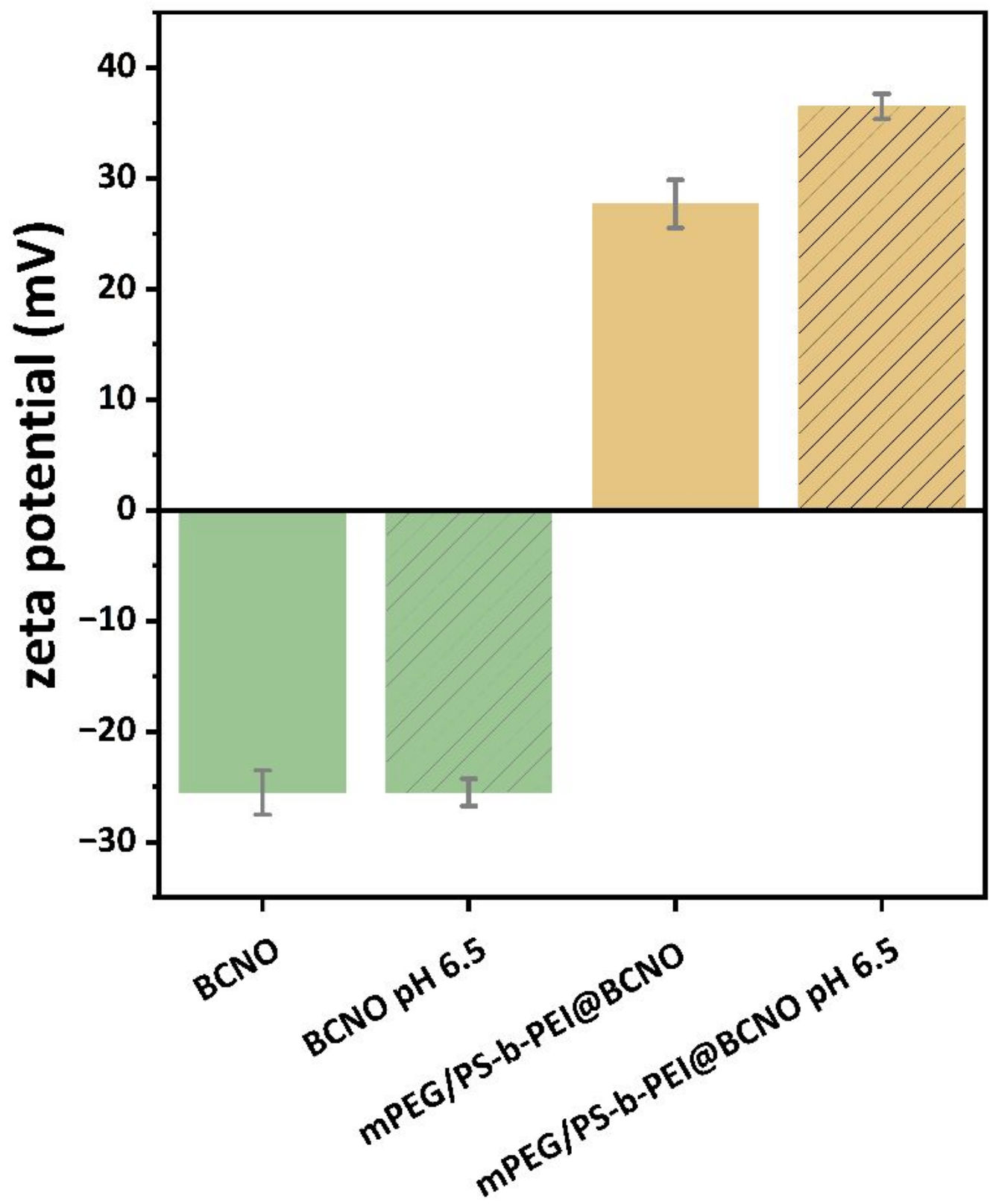

Figure S3. Zeta potential measurements of mPEG/PS-b-PEI@BCNO and BCNO before and after 9 days of incubation at pH 6.5

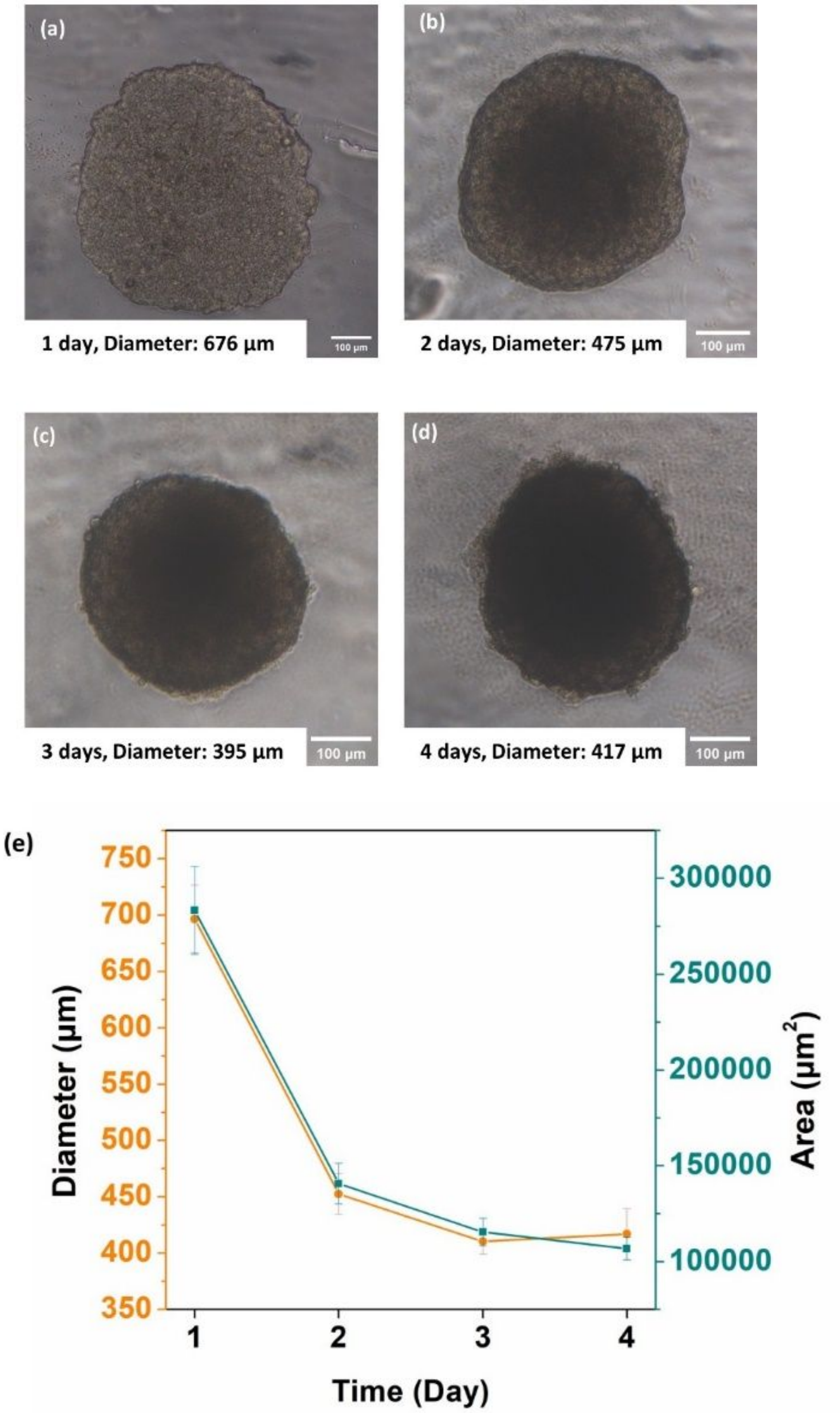

Figure S4. Representative images of UN-KC-6141 spheroids following incubation for (a) 1 day, (b) 2 days, (c) 3 days, and (d) 4 days. Scale bar: 100 µm. (e) Quantitative analysis of spheroid diameter and area was performed using Image J software.
